# Structure and Expression of Bud *Dormancy-Associated MADS-Box* Genes (*DAM*) in European Plum

**DOI:** 10.3389/fpls.2020.01288

**Published:** 2020-08-19

**Authors:** Carles Quesada-Traver, Brenda Ivette Guerrero, María Luisa Badenes, Javier Rodrigo, Gabino Ríos, Alba Lloret

**Affiliations:** ^1^Centro de Citricultura y Producción Vegetal, Instituto Valenciano de Investigaciones Agrarias (IVIA), Valencia, Spain; ^2^Unidad de Hortofruticultura, Centro de Investigación y Tecnología Agroalimentaria de Aragón (CITA), Zaragoza, Spain; ^3^Instituto Agroalimentario de Aragón-IA2, CITA-Universidad de Zaragoza, Zaragoza, Spain

**Keywords:** European plum *(Prunus domestica)*, *DAM*, bud dormancy, stress tolerance, flowering development

## Abstract

Bud dormancy in temperate perennials ensures the survival of growing meristems under the harsh environmental conditions of autumn and winter, and facilitates an optimal growth and development resumption in the spring. Although the molecular pathways controlling the dormancy process are still unclear, *DORMANCY-ASSOCIATED MADS-BOX* genes (*DAM*) have emerged as key regulators of the dormancy cycle in different species. In the present study, we have characterized the orthologs of *DAM* genes in European plum (*Prunus domestica* L.). Their expression patterns together with sequence similarities are consistent with a role of *PdoDAM*s in dormancy maintenance mechanisms in European plum. Furthermore, other genes related to dormancy, flowering, and stress response have been identified in order to obtain a molecular framework of these three different processes taking place within the dormant flower bud in this species. This research provides a set of candidate genes to be genetically modified in future research, in order to better understand dormancy regulation in perennial species.

## Introduction

Perennial species from temperate regions have to cope with seasonal changes in temperature, photoperiod, and water availability. Bud dormancy is an important adaptative mechanism ensuring survival during the cold period and paving the way for optimal growth resumption, flowering, and fruit production. During bud dormancy, these species cease growth and activate defense mechanisms, both essential to avoid injuries caused by the harmful environmental conditions during winter (Welling and Palva, 2006; Hänninen and Tanino, 2011). In European plum (*Prunus domestica* L.) and other members of the Rosaceae family, growth cessation and winter dormancy are chiefly regulated by external temperature, unlike other temperate trees that are also sensitive to photoperiod control (Heide and Prestrud, 2005; Heide, 2008). Winter dormancy presents two phases: endo- and ecodormancy (Lang et al., 1987). During endodormancy (abbreviated to dormancy in this study), the meristems remain protected within the reproductive and vegetative buds without apparent growth (Cooke et al., 2012). In this phase, flower buds require exposure to a specific range of chilling temperatures for proper flowering and subsequent fruiting. Chilling fulfilment does not cause an immediate resumption of growth because exposure to higher temperatures is also required during the phase of ecodormancy to achieve bud break and flowering. Despite the importance of distinguishing these two phases to understand the mechanisms behind dormancy, there is a lack of phenological or biological markers to detect the fulfilment of chilling (Fadón and Rodrigo, 2018). Since dormancy is highly dependent on temperature, global warming is a developing threat with potential impact on phenological transitions and fruit production. On the one hand, warmer temperatures in spring are expected to increase the risk of frosts associated with premature flowering (Luedeling et al., 2011; Guo et al., 2014; Vitasse et al., 2018; Woznicki et al., 2019). On the other hand, climatic scenarios leading to higher temperatures in winter might cause severe reductions in winter chill, becoming insufficient to fulfil chilling requirements for dormancy release and thus causing inefficient and irregular budbreak and important production losses in fruit crops (Erez, 2000; Luedeling et al., 2011; Guo et al., 2014; Legave et al., 2015). Engineering fruit crops with altered dormancy, flowering, and stress tolerance responses should enable substantial advancements in breeding time and productivity. Therefore, it is particularly important to search out the molecular factors underlying these three processes (Lloret et al., 2018).

Several studies have been focused on a group of genes, called *DORMANCY-ASSOCIATED MADS-BOX* (*DAM*) and other orthologs of *SHORT VEGETATIVE PHASE* (*SVP*) genes, which recently have emerged as potential regulators of dormancy in several species such as almond (*Prunus dulcis*; Prudencio et al., 2018), apple (*Malus x domestica*; Falavigna et al., 2014; Wu et al., 2017), apricot (*Prunus armeniaca*; Balogh et al., 2019), Chinese cherry (*Prunus pseudocerasus*; Zhu et al., 2015), hybrid aspen (*Populus tremula x tremuloides;* Singh et al., 2018), Japanese apricot (*Prunus mume*; Sasaki et al., 2011), kiwifruit (*Actinidia chinensis;* Wu et al., 2019; *Actinidia deliciosa;* Wu et al., 2012), leafy spurge (*Euphorbia esula*; Horvath et al., 2008), pear (*Pyrus pyrifolia*; Saito et al., 2015), and sweet cherry (*Prunus avium*; Rothkegel et al., 2017). These genes were firstly identified in an *evergrowing* mutant (*evg*) of peach (*Prunus persica*) that shows a non-dormant phenotype, maintaining apical growth and persistent leaves in response to dormancy inducing conditions (Bielenberg et al., 2004). This phenotype is associated with a genomic deletion that includes four out of the six tandemly-repeated *DAM* genes (Bielenberg et al., 2004; Bielenberg et al., 2008). Subsequently, other functional studies have confirmed their crucial role in the dormancy process. The ectopic expression of *DAM1* from leafy spurge in *Arabidopsis thaliana* delays bolting and flowering concomitantly with the repression of *FLOWERING LOCUS T* (*FT*) (Horvath et al., 2010). In fact, *DAM* genes have been proposed to directly repress *FT* in leafy spurge (Hao et al., 2015) and Chinese white pear (Niu et al., 2016). *PpDAM1* gene has been also described to bind and up-regulate the expression of pear *PpNCED3* gene, encoding a 9-cis-epxycarotenoid dioxygenase implicated in the synthesis of the dormancy-promoting hormone abscisic acid (Tuan et al., 2017). In addition, apple plants overexpressing *MdoDAMb* genes show delayed bud break but normal flower and fruit development (Wu et al., 2017). Finally, transgenic poplar (*Populus trichocarpa*) and apple constitutively expressing *PmDAM6* from Japanese apricot show growth inhibition and early bud set (Sasaki et al., 2011; Yamane et al., 2019). Overall, these studies suggest that *DAM* genes play a crucial role in dormancy maintenance mainly by growth and hormone regulation (Liu and Sherif, 2019; Yamane et al., 2019).

Other studies have focused on discerning the molecular mechanisms that regulate *DAM* gene expression. Some chromatin covalent modifications and miRNA have been reported to affect *DAM* expression in several species (Horvath et al., 2010; Leida et al., 2012; Saito et al., 2015; Niu et al., 2016; Vimont et al., 2020), suggesting the participation of epigenetic mechanisms in *DAM*-dependent dormancy modulation (Ríos et al., 2014; Conde et al., 2019). A TEOSINTE BRANCHED1/CYCLOIDEA/PROLIFERATING CELL FACTOR (TCP) protein (PpTCP20) binds to a specific element and down-regulates both *PpeDAM5* and *PpeDAM6* in peach (Wang et al., 2020). Also, a feedback regulatory mechanism has been described involving repression of *PpDAM1* by an abscisic acid response element (ABRE)-binding transcription factor (PpAREB1) in pear (Tuan et al., 2017). More recently, pear ABRE-BINDING FACTOR3 (PpyABF3) has been reported to specifically bind and activate *PpyDAM3* expression, whereas PpyABF2/PpAREB1 represses *PpyDAM3* by dimerising with PpyABF3 (Yang et al., 2020). Among *DAM* regulatory proteins, the most studied ones are C-Repeat Binding Factors (CBF), which are able to bind and activate *DAM* promoters by yeast one-hybrid and transient expression experiments in Japanese apricot and pear (Saito et al., 2015; Niu et al., 2016; Zhao et al., 2018b). Interestingly, CBF factors are also involved in the cold-temperature response pathway (Wisniewski et al., 2011; Wisniewski et al., 2018), revealing the close relationship between dormancy and low temperature tolerance mechanisms.

Plants reprogram their gene expression profile in order to cope with cold and desiccation stresses associated with the dormancy period. Thus, the analysis of differentially expressed genes in dormant buds has provided many candidate genes belonging to abiotic tolerance responses. The ectopic expression of the cold acclimation response gene *CBF* from peach improves freezing tolerance in apple (Wisniewski et al., 2011; Wisniewski et al., 2015). Several studies suggest that *evg* mutant of peach has lower cold tolerance due in part to a lack of a dehydrin accumulated in bark tissues during dormancy progression (Arora et al., 1992; Arora and Wisniewski, 1994; Arora et al., 1996; Artlip et al., 1997). In addition, the ectopic expression of *STRESS-ASSOCIATED PROTEIN1* (*PpSAP1*) gene, highly expressed in dormant buds of peach, improves water retention under desiccation conditions in transgenic plum (Lloret et al., 2017a). Moreover, some genes related to sugar metabolism pathways with altered expression along bud development have been shown to participate in tolerance mechanisms. In peach, *PpeS6PDH* gene encoding a sorbitol-6-phosphate dehydrogenase has been postulated to synthesize the compatible solute sorbitol in order to protect dormant buds against cold and hydric stresses (Lloret et al., 2017b). In addition, galactinol synthase (GolS) genes, involved in the synthesis of raffinose family oligosaccharides, are up-regulated in dormant buds of chestnut and apple (Ibáñez et al., 2013; Falavigna et al., 2018). Interestingly, the overexpression of apple *MdGolS2* confers tolerance to water deficit in *Arabidopsis* (Falavigna et al., 2018).

During winter dormancy, growth and development of flower organs ceases in order to deal with the harmful environmental conditions. In stone fruits, flower bud formation takes place in summer in the year preceding flowering and fruiting, whereas flower primordia stop growing and remain dormant inside the buds. The dormancy arrest guarantees an optimal development of gametes under more suitable conditions after dormancy release, which associates with a sharp up-regulation of genes involved in pollen microsporogenesis, among other flowering processes (Ríos et al., 2013).

European plum is the fruit tree species with the highest number of cultivars in Europe (Esmenjaud and Dirlewanger, 2007). Among them, ‘Reine Claude Verte’ is the most grown cultivar for its excellent organoleptic qualities (Gharbi et al., 2014). This cultivar has been cultivated for more than 500 years in Europe, from where it has expanded worldwide (Tabuenca and Iturrioz, 1991). European plum is also a genetic model for other *Prunus* species by virtue of the availability of efficient procedures for genetic transformation and regeneration (Petri et al., 2018). Based on previous studies in peach and other species, this study aims at identifying and characterizing the *DAM* and other dormancy related genes from European plum, in order to obtain a dynamic snapshot of molecular mechanisms and factors affecting dormancy in flower buds of this species. This research will open the possibility to initiate functional studies on dormancy related genes with the use of transgenic plum plants overexpressing or down-regulating some of these genes.

## Results

### Chilling Requirements for Dormancy Release in European Plum

**Figure 1:**
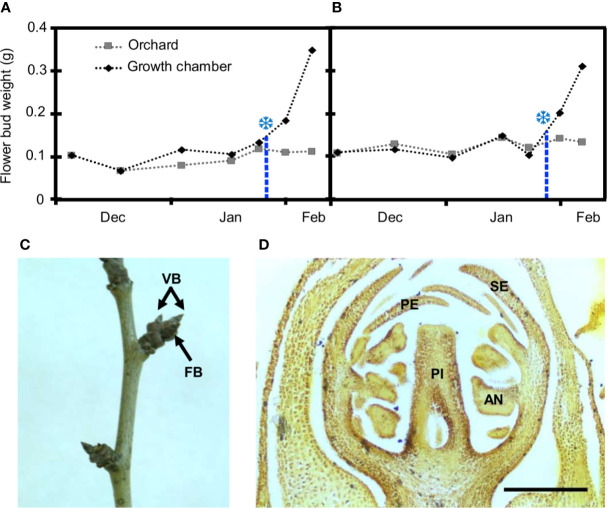
Estimation of breaking of dormancy and flower bud characterization in European plum cv. ‘Reine Claude Verte.’ Flower bud weight in orchard conditions (gray squares) and after 8 days in the growth chamber (black rhombi) over two seasons: 2018–2019 **(A)** and 2019–2020 **(B)**. Snowflakes mark the weight increment of 30%, when chilling was fulfilled. **(C)** Shoot of European plum cv. ‘Reina Claude Verte’ during dormancy, showing flower (FB), and vegetative (VB) buds. **(D)** Longitudinal section of a flower primordium at the date of dormancy breaking. Sepals (SE), petals (PE), anthers (AN), and pistil (PI); scale bar, 300 µm.

The chilling requirements for dormancy release of flower buds from European plum cv. ‘Reine Claude Verte’ were estimated during two consecutive years. The annual temperature regime was very similar in both years, showing a close pattern of chilling accumulation (**Supplementary Figure S1**). Chilling fulfilment was achieved at similar dates in both seasons, January 25 for 2018–2019 and January 27 for 2019–2020 (**Figures 1A, B**). The date of chilling fulfilment allowed estimating the chilling requirements of ‘Reine Claude Verte’ by the calculation of the number of chilling hours (CH), chilling units (CU), and chilling portions (CP) accumulated until then. The range of the values obtained in the two seasons, 979–1,086 CH, 1,248–1,287 CU, and 62.8 CP (same value in both years), were considered as the chilling requirements of this cultivar (**Supplementary Table S1**).

From the end of autumn and during winter, all the flower buds were closed and covered by dark brown scales at phenological stage A (Baggiolini, 1952) and stage BBCH 50 (Fadón et al., 2015) (**Figure 1C**). At this stage, two to three flower primordia were present inside each flower bud with all the whorls (sepals, petals, anthers, and pistil) differentiated (**Figures 1D**). In autumn, the average weight of flower buds was around 0.1 g, and it remained without significant variations throughout dormancy in both years (**Figures 1A, B**). No external phenological changes were observed until bud burst in spring several weeks after chilling fulfilment.

### Genome-Wide Identification of *PdoDAM1-6* Genes From European Plum

**Figure 2:**
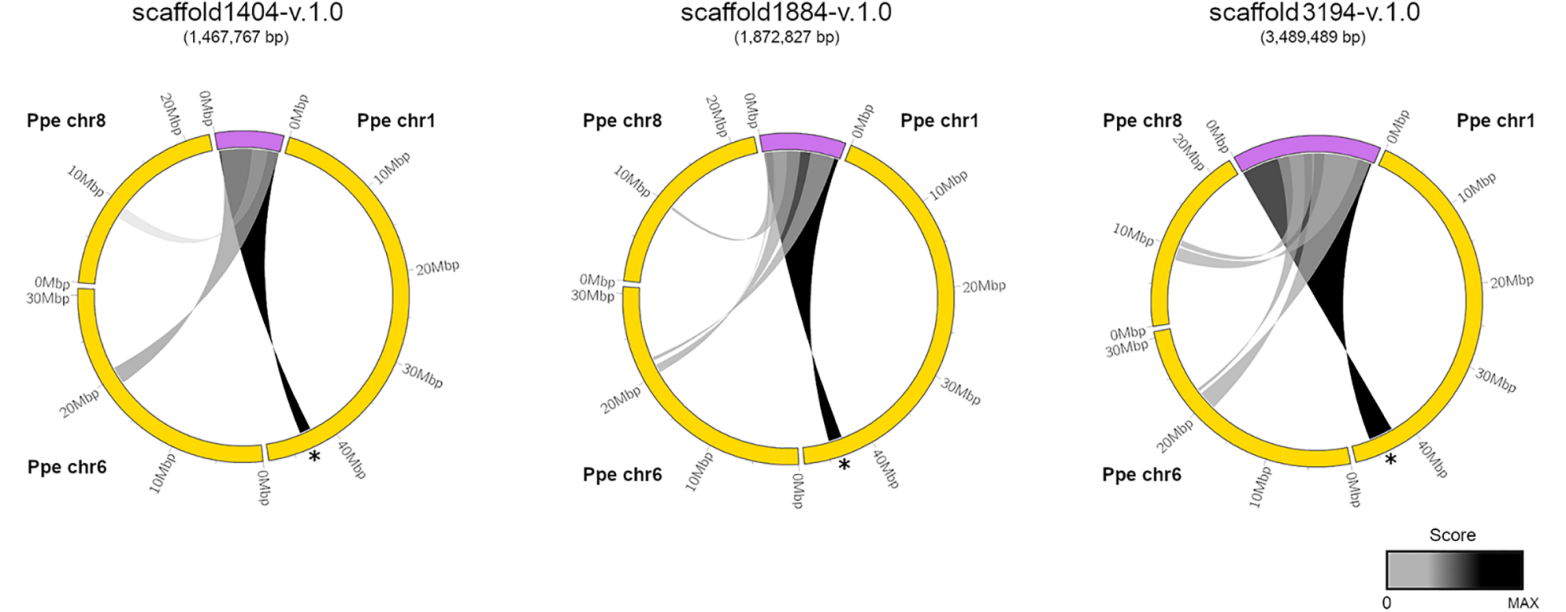
Syntenic relationships between European plum scaffolds containing the *DAM* locus and the peach genome. European plum scaffold1404-v.1.0, scaffold1884-v.1.0, and scaffold 3194-v.1.0 (purple) and peach chromosomes Ppe chr1, Ppe chr6, and Ppe chr8 (yellow) are represented using ClicO FS. Scoring regions are connected by a ribbon and colored according to a grayscale that associates black color to the maximum score for each representation. *DAM* region in Ppe chr1 is marked with a black asterisk. European plum scaffolds are five-fold magnified in the representation for a proper visualization.

We used the coding sequence of *PpeDAM1-6* genes from peach (*Prunus persica*) for a BLASTN search in the *Prunus domestica* v1.0 draft genome, recently uploaded to the Genome Database for Rosaceae (GDR; https://www.rosaceae.org/). We found three assembled scaffolds that exhibited high similarity to *DAM* locus of peach and contained at least the putative orthologues of the six *PpeDAM* genes and their flanking genes. The circle plot revealed strong conservation along the three scaffolds with the *PpeDAM* region, localized in the chromosome 1 of peach (**Figure 2**). However, they also presented a weaker synteny with peach chromosomes 6 and 8. In this region of the chromosome 6, we found the putative ortholog in the peach genome of *Arabidopsis SHORT VEGETATIVE PHASE* (*SVP*), systematically named Prupe.6G199000, belonging to the *SVP*/StMADS11 lineage of type II MIKC^C^ MADS-box genes in which *DAM* genes are also clustered (Jiménez et al., 2009). On the other hand, the syntenic region in chromosome 8 was quite divergent and, in fact, did not include any *DAM-*like gene.

**Figure 3:**
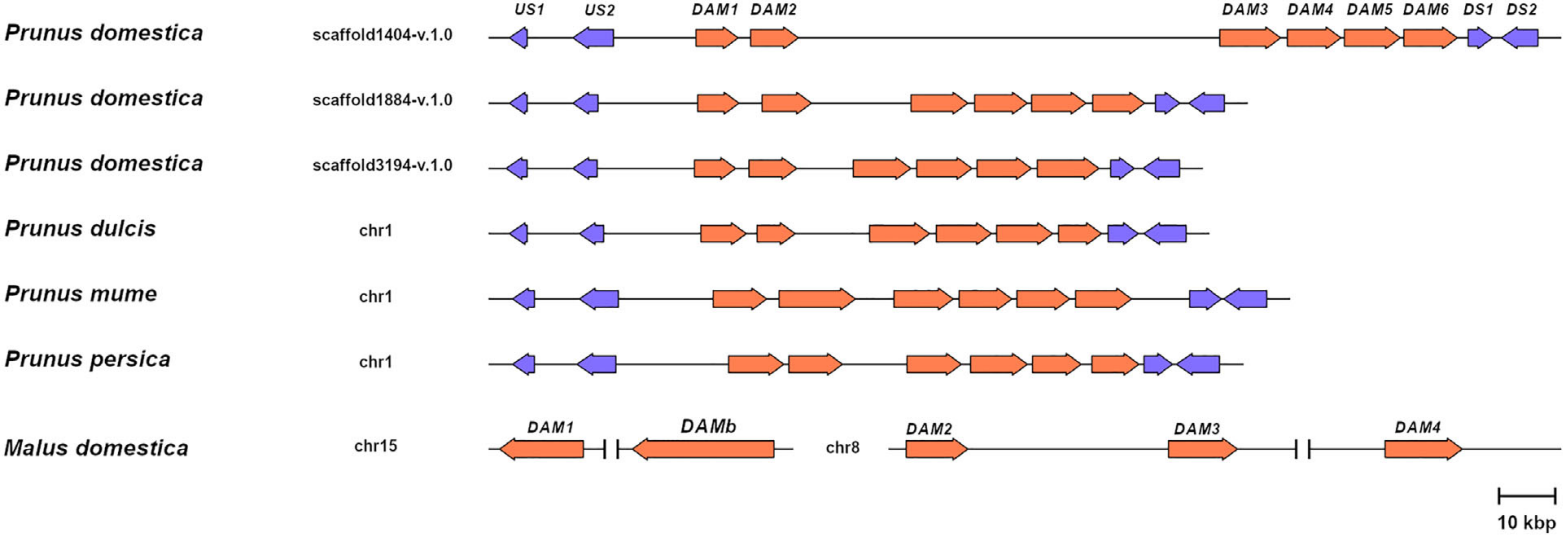
Relative genomic representation of *DAM* loci from different species. *DAM* genes (orange) and the closest two upstream (US1-2) and downstream genes (DS1-2) in *Prunus* (blue). Apple *MdDAM1-4* and *MdDAMb* are also represented. The scale bar represents a 10 kbp genomic distance.

**Figure 4:**
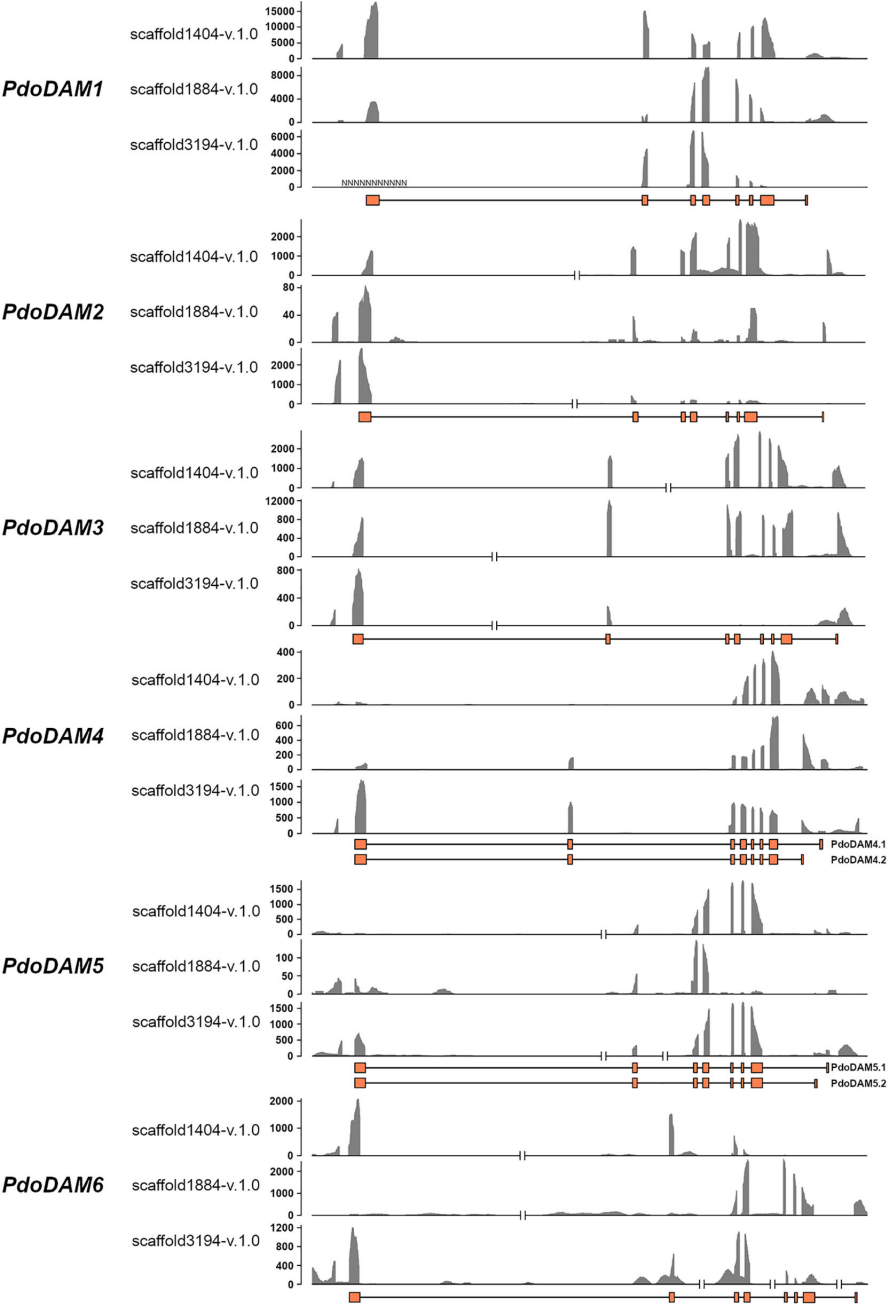
RNA coverage representation in *DAM* genes for each European plum scaffold. For each *DAM* gene, three coverage graphs corresponding to the different scaffolds represent number of reads. The predicted exonic structure is depicted below (orange boxes). The coverage plot was obtained by merging bam files from three RNA samples.

To support the conservation of the *DAM* genomic region in European plum, we analyzed in detail the organization of the putative *DAM* genes and their genomic neighborhood. As shown in **Figure 3**, *PdoDAM* genes preserved the gene order observed in peach and other *Prunus* species, and the two closest downstream and upstream flanking genes were also present in the three scaffolds under study. On the contrary, *DAM* genes localization was more fragmented in apple. Interestingly, the *DAM* loci identified in the three scaffolds of European plum were different in length, mainly caused by a variable intergenic region between putative *PdoDAM2* and *PdoDAM3* genes. This variability could be due to the fact that polyploid species such as *Prunus domestica*, which has a hexaploid genome, in addition to having six different alleles, have a much more plastic genome structure than their progenitor diploids (Leitch and Leitch, 2008; Fu et al., 2016). In order to identify the transcript sequence of *PdoDAM* genes, RNA-seq data derived from leaves of European plum cv. ‘Reine Claude Verte’ were used for gene prediction. These transcriptomic data were obtained in our laboratory and uploaded to NCBI BioProject database (ID PRJNA630876). The mRNA coverage plot of each scaffold was merged in order to obtain a more reliable predicted sequence. All six *PdoDAMs* had similar gene structures consisting of eight exons and seven introns flanked by the translation initiation and stop codons (**Figure 4**). Transcriptomic data suggested an alternative splicing for *PdoDAM4-*like and *PdoDAM5-*like, based on RNA-seq data and the presence of stop codons at the 3’ end. Both transcripts were confirmed by gene expression analyses during bud development although *PdoDAM4.1* and *PdoDAM5.2* were clearly less abundant (**Supplementary Table S2**). As the exons were well conserved across the three scaffolds (**Supplementary Table S3**), we selected *PdoDAMs* predicted coding sequences from scaffold1404-v.1.0 and the most abundant isoform in each case for subsequent analysis and used the names *PdoDAM1-6* to designate them (**Supplementary Figure S2**).

**Figure 5:**
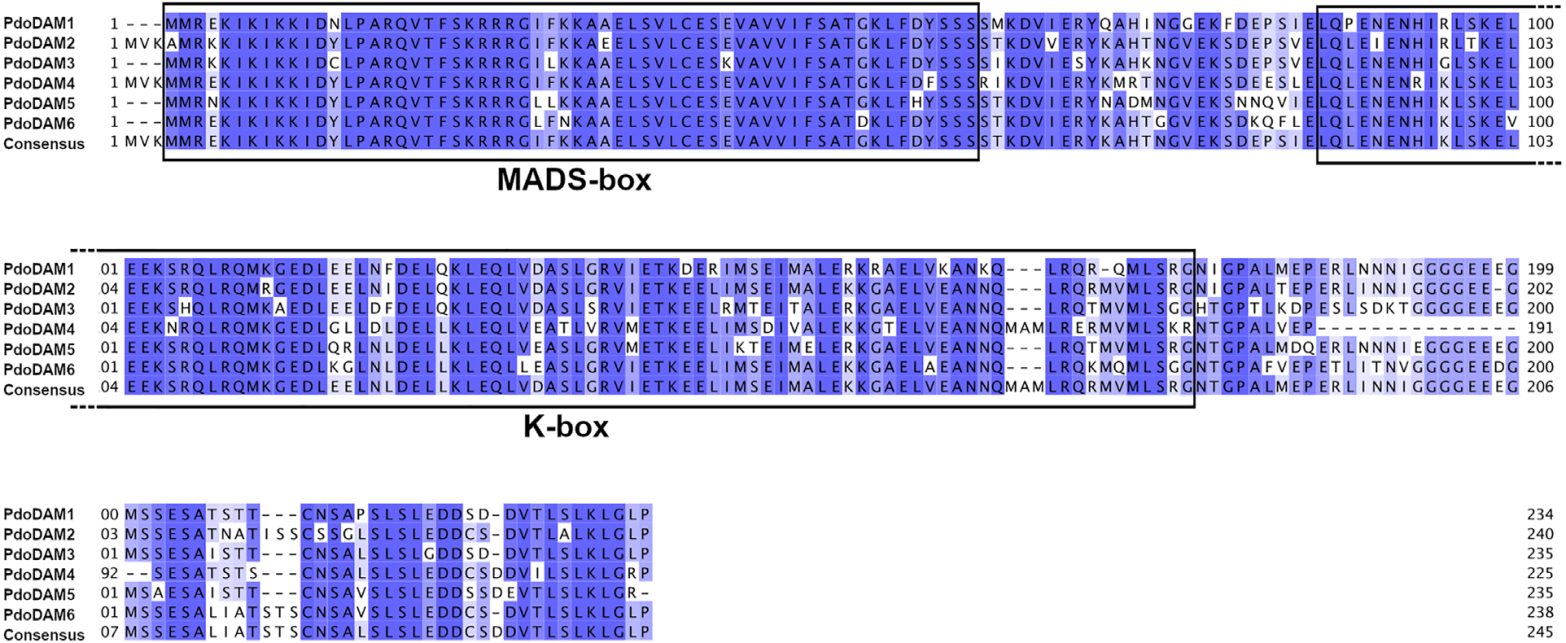
Alignment of predicted PdoDAM1-6 protein sequences. MADS-box and K-box domains are highlighted. Alignment was performed using MUSCLE algorithm. Colored positions represent homology between sequences.

The deduced amino acid sequences of all identified *PdoDAMs* contained the highly conserved DNA binding MADS-box domain at the N-terminal end, the K-domain participating in protein-protein interactions and finally the variable intervening region that connects both domains (**Figure 5**). These observations confirmed that *PdoDAM* genes belong to the MIKC^c^-type of MADS-box genes, consistently with previous studies in other species (Jiménez et al., 2009; Zhao et al., 2018a).

**Figure 6:**
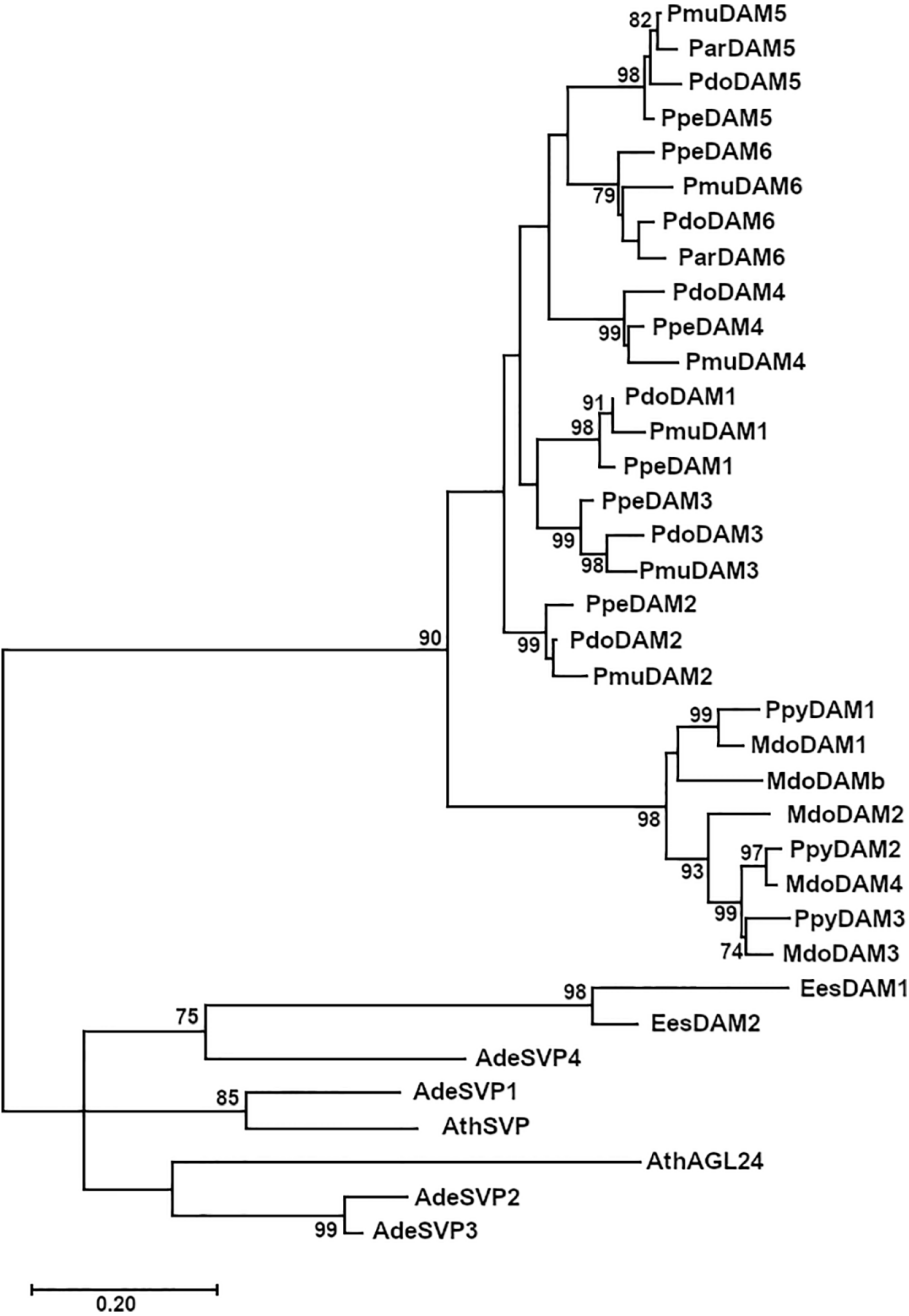
Phylogenetic tree of PdoDAM1-6 and other DAM-like proteins. DAM sequences from *Prunus domestica* (European plum, “Pdo”), *Prunus mume* (Japanese apricot, “Pmu”), *Prunus armeniaca* (apricot, “Par”), *Prunus persica* (peach, “Ppe”), and *Malus domestica* (apple, “Mdo”) along with related protein sequences from *Pyrus pyrifolia* (pear, “Ppy”), *Arabidopsis thaliana* (“At”), *Actinidia deliciosa* (kiwifruit, “Ade”), and *Euphorbia esula* (leafy spurge, “Ees”). The Maximum Likelihood method was used to construct the tree and it was bootstrapped 1,000 times. The branch length corresponding to the number of substitutions per amino acid is represented in the scale bar.

The phylogenetic analysis of PdoDAM1-6 predicted protein sequences indicated that they form part of a differentiated group in conjunction with other *Prunus* sequences, which was divided into six subgroups corresponding to the six tandemly repeated *DAM* genes (**Figure 6**). In addition, DAM proteins from genera *Malus* and *Pyrus* constituted another well-defined group, that jointly with *Prunus* DAMs were clearly differentiated from a cluster composed by SVP-like proteins from *Arabidopsis thaliana* and kiwifruit (*Actinidia deliciosa*) and EeDAM1-2 from leafy spurge.

### Expression Analysis of *PdoDAM1-6* Genes

**Figure 7:**
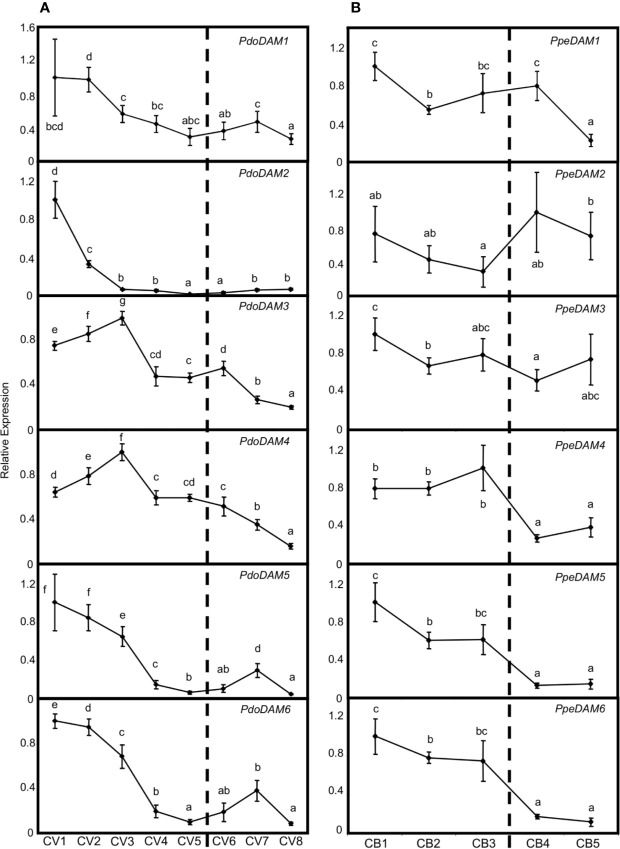
Relative expression of *DAM* genes in flower buds. *PdoDAM1-6* gene expression in European plum cv. ‘Reine Claude Verte’ (CV) is represented in panel **(A)**, and *PpeDAM1-6* gene expression in peach cv. ‘Crimson Baby’ (CB) in panel **(B)**. Timepoint codes are found in *Material and Methods*. The dashed bar indicates dormancy release. For each graph, an expression of one is assigned to the highest value. Each point represents data of three biological replicates accompanied by error bars representing its standard deviation. Significant differences among samples are represented by different letters (a–e), with a confidence level of 95%.

Since the best-known function of previously described *DAM*-like genes has been related to bud dormancy regulation, we measured *PdoDAM1-6* gene expression across bud development in European plum cv. ‘Reine Claude Verte’ during the winter of 2018/2019. The six *PdoDAM1-6* genes showed a general progressive decrease in mRNA level until the dormancy period was completely overcome, with specific gene particularities (**Figure 7A**). Despite *PdoDAM1* expression did not show great changes along bud development, a slight down-regulation was observed from the first two samples, collected in autumn. On the contrary, *PdoDAM2* was the European plum *DAM* gene with strongest gene down-regulation, occurring in early stages before dormancy release (November–December), and maintained along the whole bud development process. *PdoDAM3* and *PdoDAM4* presented a similar expression pattern, with transcripts levels increasing slightly from November to December and then declining during bud dormancy progression. Finally, *PdoDAM5* and *PdoDAM6* also showed paralleled expression profiles. They were strongly down-regulated prior to bud dormancy release, and subsequently they slightly peaked in ecodormant CV7 sample, at the beginning of February.

When comparing these expression patterns with those of *PpeDAM* genes in peach cv. ‘Crimson Baby’ at five different bud dormancy samples (CB1–CB5), we found both common and specific features (**Figure 7B**). Whereas *PpeDAM1*, *PpeDAM2*, and *PpeDAM3* showed a quite different expression profile from their European plum counterparts, *PpeDAM4*, *PpeDAM5*, and *PpeDAM6* reduced their expression during bud development in a broadly similar fashion to European plum orthologs. *PpeDAM4* slightly increased its expression level in the first samples, followed by a sharp down-regulation concomitantly with dormancy release. As previously described, *PpeDAM5* and *PpeDAM6* transcript levels decreased in precise concordance with bud dormancy release. Interestingly, plum *PdoDAM4*, *PdoDAM5*, and *PdoDAM6* gene repression occurred similarly but in a slightly more advanced manner than in their peach orthologs.

### Analysis of Cis-Regulatory Elements in *PdoDAM1-6* Regulatory Regions

**Figure 8:**
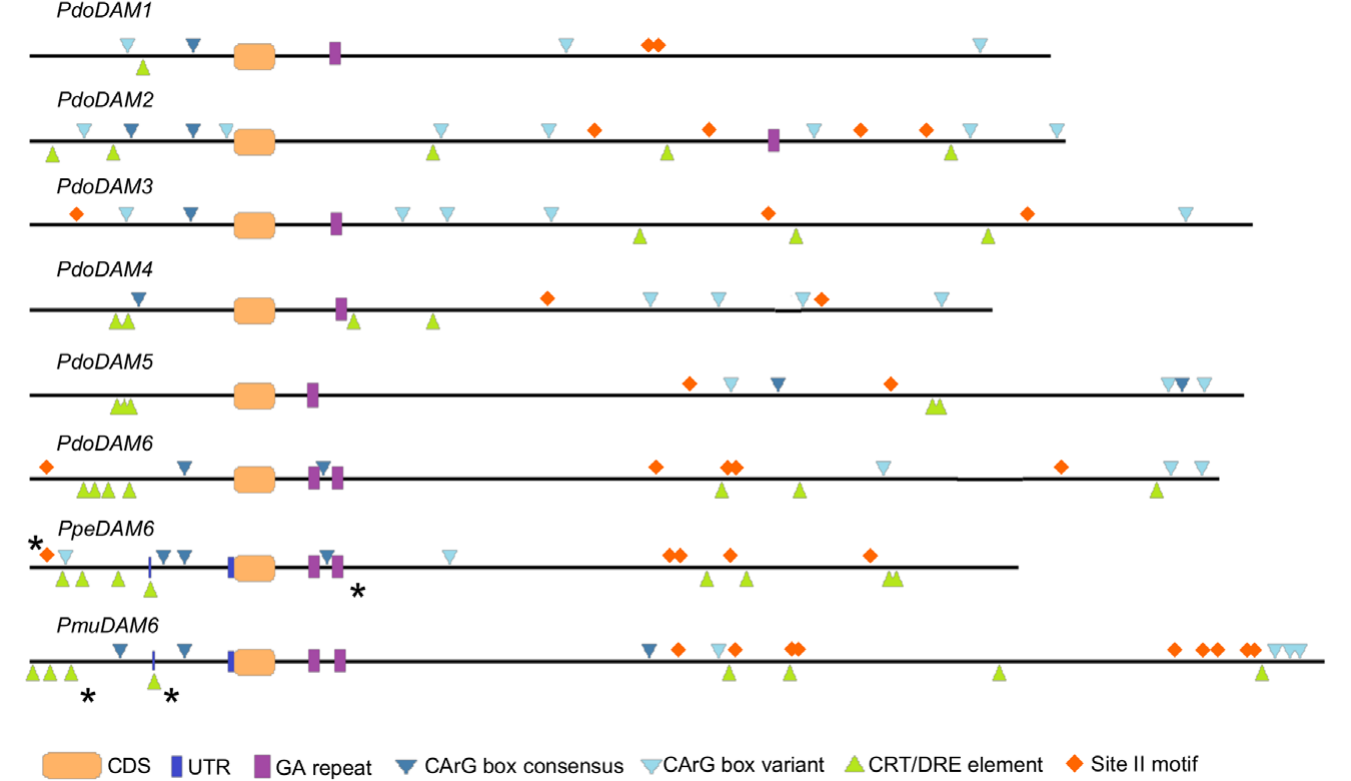
Cis-regulatory elements in the promoter and first intron of *DAM* genes. Promoters and introns of European plum *PdoDAM1-6*, peach *PpeDAM6*, and Japanese apricot *PmuDAM6* are represented by black lines interrupted by exonic sequences (orange rectangles). The potential cis-regulatory elements GA repeat (purple boxes), CArG box consensus (dark blue triangles) and variant (light blue triangles), CRT/DRE element (green triangles), and site II motif (orange rhombi) are labeled. Regulatory elements confirmed in previous experimental studies are marked with a black asterisk.

We searched in *PdoDAM* genes different motifs described in previous reports as regulatory elements of *DAM* genes from other species. Since these elements were previously identified in both, the promoter and the intronic region between the first exons of *DAM* genes, we focused on those regulatory genomic regions in *PdoDAM* genes. MADS-box transcription factors bind DNA sequences known as CArG box motif with the consensus sequence CC(A/T)_6_GG or the non-canonical C(A/T)_8_G. MADS-box genes, as *DAM* genes, have been reported to interact with these elements to regulate the expression of other genes but also to regulate themselves (Zhu and Perry, 2005; Gregis et al., 2013). The presence of CArG box sequences in all *PdoDAM* genes is thus consistent with self-regulation mechanisms (**Figure 8**). On the other hand, CBF proteins play a critical role in activation of cold responsive genes. *PmuDAM6* gene was reported to be transcriptionally regulated by the direct binding of PmuCBFs to C-repeat/dehydration-responsive elements (CRT/DRE; Zhao et al., 2018b). As shown in **Figure 8**, these elements were identified along the whole genomic regions under study, but were particularly abundant on the promoters of *DAM6* genes from Japanese apricot, peach, and European plum, in a position close to active CRTs described by the literature. We also highlighted the site II motif associated with the sequence GCCCA. This element is recognized by TCP transcription factors described as down-regulators of *PpeDAM5* and *PpeDAM6* expression in peach (Wang et al., 2020). Site II motifs were identified in the intronic regions of all six *PdoDAM* genes and the promoters of *PdoDAM3* and *PdoDAM6*, closely located to the one reported by the literature.

### Expression Analysis of Other Dormancy-Related Genes

**Figure 9:**
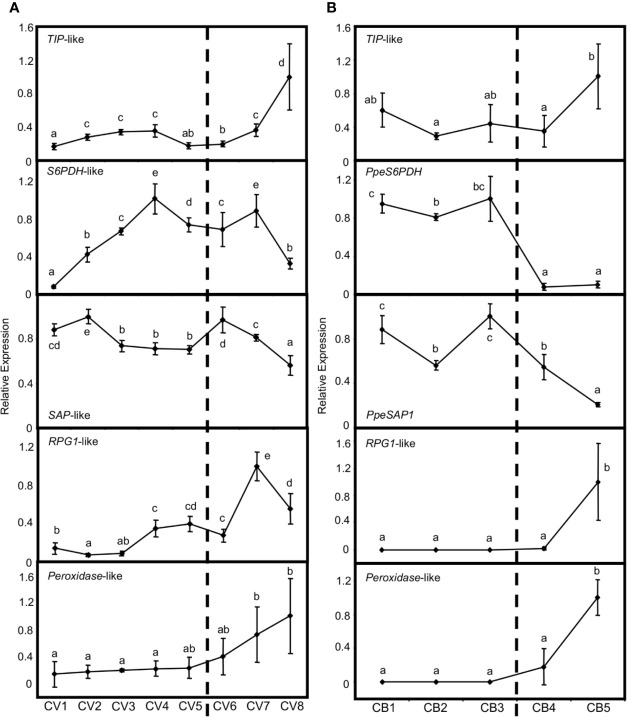
Relative expression of dormancy-related genes in flower buds. Bud samples from European plum cv. ‘Reine Claude Verte’ (CV) and peach cv. ‘Crimson Baby’ (CB) are respectively represented in panels **(A, B)**. Timepoint codes are found in *Material and Methods*. The dashed bar indicates dormancy release. For each graph, an expression of one is assigned to the highest value. Each point represents data of three biological replicates accompanied by error bars representing its standard deviation. Significant differences among samples are represented by different letters (a–e), with a confidence level of 95%.

Previous genome-wide transcriptional analyses have provided numerous genes differentially expressed in flower buds of peach, which are related to three major coincident processes: bud dormancy regulation, stress tolerance and flowering development (Ríos et al., 2013; Lloret et al., 2017a, Lloret et al., 2017b). We analyzed the bud-dependent expression of the putative orthologs in European plum of some of these genes. Firstly, a *TONOPLAST INTRINSIC PROTEIN* (*TIP*)-like gene increased its expression in flower buds of European plum after dormancy release (**Figure 9A**), in close agreement with the behavior of its peach ortholog and its proposed function in tonoplast turgor and growth resumption (**Figure 9B**). We also studied the expression patterns of *SORBITOL-6-PHOSPHATE DEHYDROGENASE* (*S6PDH*)-like and *STRESS ASSOCIATED PROTEIN* (*SAP*)-like genes, which are postulated to participate in the stress tolerance response during bud dormancy in peach. Interestingly, *S6PDH*-like increased its expression level during dormancy progression until January 16^th^ (CV4) and from that point it became steady and started to decrease. On the other hand, *SAP-*like expression was quite stable along dormancy progression and decreased after dormancy release (**Figure 9A**). Finally, *RUPTURED POLLEN GRAIN1* (*RPG1*)*-*like and peroxidase-like genes, associated with microsporogenesis and pollen development in anthers, were up-regulated after the dormancy period, coincidently with the last phases of flower development usually activated at this stage. The expression profile of these genes was in concordance with peach bud expression patterns (**Figure 9B**).

## Discussion

The susceptibility of phenological transitions to changing climatic scenarios is a major challenge for temperate fruit crops, with a high potential impact on flowering and fruit production, as was already reported for European plum (Woznicki et al., 2019). Among these phenological transitions, bud dormancy modulates consecutively growth cessation and resumption in response to seasonal environmental conditions, and directly affects flowering timing and efficiency, which makes the understanding of this process essential for improving adaptation strategies to global temperature changes. The estimation of chilling requirements for bud dormancy release, based on bud-break forcing assays in combination with chilling quantification using one or more mathematical models, has become a key tool for the estimation of adaptability of a species or cultivar. In particular, chilling requirement of European plum cv. ‘Reine Claude Verte’ calculated in this study (979–1,086 CH) is in close agreement with previous estimations (976–1,275 CH; Tabuenca, 1967), and similar to those reported in other European plum cultivars (Fadón et al., 2020).

Bud dormancy is regulated by *DAM* transcription factor genes, mainly in *Prunus* species (Bielenberg et al., 2008; Sasaki et al., 2011; Balogh et al., 2019). The availability of the draft genome of European plum, recently uploaded to the GDR database, has allowed the identification and genomic characterization of six plum *DAM* genes in this study, designated *PdoDAM1-6*. Domain alignments and phylogenetic analysis indicated that they are closely related to the corresponding *Prunus DAM* sequences. *PdoDAM* genomic regions showed high synteny with three different regions of the peach genome. The strongest synteny corresponds to the end of chromosome 1 where *PpeDAM* are located. However, the second syntenic block is situated within chromosome 6, containing the putative ortholog of *SVP* in peach (*PpeMADS57*), as stated in a systematic analysis of the MADS-box family in peach (Wells et al., 2015). This observation showed the relationship between *PdoDAM* genes and *SVP* genes, as has been already reported in peach (Jiménez et al., 2009). On the other hand, the expansion of *DAM* genes has been proposed to be originated by serial tandem duplications before the diversification of the *Prunus* genus (Jiménez et al., 2009; Zhang et al., 2012), consistently also with this study. Expansion of *DAM* genes occurred independently in the Maleae tribe including apple and pear, as supported by the identification of several *DAM*-like genes with a common genomic organization, essentially different from *Prunus* species (Wu et al., 2017). The independent origin of these duplication events and the observation of a strong purifying selection in all six *PpeDAM* genes, in spite of their sequence similarity, suggest the specialization of these genes for unique roles (Jiménez et al., 2009).

This proposed neo-functionalization of *DAM* genes is coherent with their different expression timing during dormancy progression. In this respect, our gene expression data in European plum matched previous studies in other related species. Our results showed an increase in *PdoDAM4* expression levels from November to December, followed by gene down regulation prolonged after dormancy release, as similarly reported in other *Prunus* species (Leida et al., 2010; Yu et al., 2020). On the other hand, peach *PpeDAM5* and *PpeDAM6* expression were inversely correlated with dormancy release, and thus were considered good predictors of the dormancy stage of buds (Li et al., 2009; Jiménez et al., 2010; Leida et al., 2010; Leida et al., 2012). European plum *PdoDAM5* and *PdoDAM6* showed a similar transcript level decrease, although it occurred earlier than in peach, using the estimated date of dormancy release as reference. Once dormancy was released, both *PdoDAM5* and *PdoDAM6* profiles reached a secondary minor peak, which was similarly reported in apricot *ParDAM5* and *ParDAM6* genes (Balogh et al., 2019). In spite of their transcriptional particularities, *PdoDAM* genes were in general down-regulated along bud dormancy and development, according to previous studies (Sasaki et al., 2011; Zhao et al., 2018a; Yu et al., 2020).

Gene expression diversification and neo-functionalization of duplicated *DAM* genes could be at least partially originated in the variation of regulatory elements responding to different, but closely related environmental signals (Haake et al., 2002; Lawton-Rauh, 2003) both in promoters and in first kilobases of intronic regions (Rose, 2019). The identification of chromatin modifications related to gene activity and silencing in the large intron of *DAM* genes in peach (Leida et al., 2012; de la Fuente et al., 2015) recommends the search of intronic sequences in addition to promoters. The direct binding of CBF proteins to CRT/DRE elements in promoter regions has been proposed to mediate the effect of low temperature on *DAM* gene expression in leafy spurge (Horvath et al., 2010), Japanese pear (Saito et al., 2015; Niu et al., 2016), apple (Mimida et al., 2015; Wisniewski et al., 2015), and Japanese apricot (Zhao et al., 2018a). The distinct number and position of these CRT/DRE elements on the specific *PdoDAM1-6* promoters are expected to affect their particular environmental response. Other transcription factors involved in regulation of *DAM* genes in other species have also cis-regulatory elements in the promoter and large intron of *PdoDAMs*. Among them, site II motifs are able to bind TCP factors similar to PpeTCP20, which is involved in *PpeDAM5* and *PpeDAM6* down-regulation when dormancy is released in peach (Wang et al., 2020). On the other hand, MADS-box factors binding to CArG box sequences are in certain cases self-regulating their own expression (Zhu and Perry, 2005; Gregis et al., 2013), and thus we cannot discard the cross-regulation of *DAM* genes in complex with other DAM or MADS-box proteins, as was indirectly suggested by the down-regulation of the whole *DAM* array of genes when four of them were deleted in the *evg* mutant of peach (Bielenberg et al., 2008). In addition to conventional transcription factors, also chromatin modification complexes are affecting *DAM* genes (Horvath et al., 2010; Leida et al., 2012; Saito et al., 2015; Vimont et al., 2020). Conserved GA repeat sequences have been found associated with regions enriched in the H3K27me3 histone modification in introns of *PpeDAM* genes (de la Fuente et al., 2015), other peach dormancy-regulated genes (Lloret et al., 2017b), and *Arabidopsis* genes (Deng et al., 2013). These GA repeats were found in a similar position and structure in *PdoDAM* genes, which suggests the conservation of chromatin-dependent mechanisms involved in *DAM* silencing responding to seasonal chilling accumulation (Ríos et al., 2014). In addition to DAMs, other genes have been postulated to participate in dormancy regulation and other concurrent processes which are precisely orchestrated within flower buds, that is cold and hydric stress tolerance and flowering pathways (Lloret et al., 2018).

We identified putative orthologs of some of these genes in European plum. One of them encoded a TIP aquaporin involved in water permeability and transport of small molecules across the tonoplast membrane. *TIP*-like genes have been related to cell turgor and cell enlargement (Ludevid et al., 1992; Maurel et al., 2015). *TIP*-like gene was induced once dormancy was released in both peach and European plum, supporting a role in growth resumption and bud-break in ecodormant buds (Lloret et al., 2017a). Interestingly, the transport of metabolites and regulatory molecules across cell membranes has been found related to cell-to-cell communication and bud dormancy regulation in previous articles (Rinne et al., 2001; Rinne et al., 2011; Tylewicz et al., 2018). Meristem cells are isolated by plasmodesmata callose plugging during dormancy, and exposure to chilling temperatures causes the degradation of the callose and the subsequent restoration of cell-to-cell communication. This mechanism is based on the action of 1-3-ß-D-glucanases (Rinne et al., 2001) and abscisic acid (ABA) (Tylewicz et al., 2018). Three groups of genes, members of the *GLUCAN HYDROLASE 17* (*GH17*) family, are upregulated by the chilling temperatures and biosynthesis of gibberellins. Short-term photoperiodic exposure transiently regulates the group 1 *GH17* genes to maintain the symplasmic paths to facilitate bud formation. Gibberellin GA_3_ and long-term chilling exposure up-regulate the groups 2 and 3 *GH17* genes, allowing callose removal and reopening the signaling. After the required chilling accumulation, mild temperatures up-regulate growth-related genes, mediated by GA_4_, leading to bud burst (Rinne et al., 2011). The tonoplast localization of TIP protein precludes its participation in these cell-to-cell communication processes, however it supports the key role of membrane permeability in dormancy release events.

On the other hand, two genes described as stress tolerance factors during peach dormancy, *S6PDH*-like and *SAP1*-like were also differentially regulated during bud dormancy in European plum. *PpeS6PDH* codifies a sorbitol-6-phosphate dehydrogenase involved in sorbitol synthesis, a major translocatable photosynthate in Rosaceae species that has been hypothesized to act as a compatible solute protecting dormant buds against cold and hydric stresses (Lloret et al., 2017b). Furthermore, the ectopic expression of *PpeSAP1* altered leaf morphology and reduced water loss in transgenic plums (Lloret et al., 2017a). The expression profile of *S6PDH*-like and *SAP1*-like genes suggests the conservation of their respective protective roles against the harmful environmental conditions in dormant buds of different *Prunus* species. Finally, we also identified genes related to flowering pathways previously reported to be differentially expressed in peach (Ríos et al., 2013). A peroxidase-like and *RPG1*-like genes are involved in sporopollenin synthesis, a component of the outer cell wall of the pollen grain (exine) essential for pollen maturation and viability (Guan et al., 2008; Jacobowitz et al., 2019). These genes were up-regulated after flower bud dormancy release in peach and European plum in this work, as expected for genes participating in pollen development in the tapetum layer of anthers, since this process is finely coordinated with the dormancy period (Julian et al., 2011).

This work provides putative key elements of a molecular framework of bud dormancy regulation and other concurrent processes lying in flower buds of European plum. This species has efficient procedures for genetic transformation (Petri et al., 2018), as opposed to most woody fruit species, which are recalcitrant for genetic transformation and regeneration (Petri and Burgos, 2005). Thus, genes identified in this work are potential candidate genes for studying dormancy regulation and other related processes by RNA interference and other functional genetics procedures.

## Material and Methods

### Plant Material and Sample Collection

Six trees of European plum (*Prunus domestica* L.) cv. ‘Reine Claude Verte’ were selected for the experiment from a collection held at the Centro de Investigación y Tecnología Agroalimentaria, CITA, Zaragoza, Spain, at 41°44´30” N, 0°47´00” W, and 220 m above sea level. For flower bud development expression analysis, buds were collected during autumn–winter 2018–2019 on November 28 (CV1), December 5 (CV2), December 18 (CV3), January 16 (CV4), January 23 (CV5), January 30 (CV6), February 6 (CV7), and February 13 (CV8).

Peach trees (*Prunus persica* L. Batsch cv. ‘Crimson Baby’) required in this study were grown at the Instituto Valenciano de Investigaciones Agrarias located in Moncada, Spain, at 39°35’20” N, 0°23’43” W, and 75 m above sea level. For expression analysis, flower buds were collected during autumn–winter 2015–2016 on November 30 (CB1), December 14 (CB2), December 21 (CB3), January 4 (CB4), and January 19 (CB5).

### Determination of Breaking of Dormancy

Dormancy was experimentally determined for ‘Reine Claude Verte’ over two consecutive winters (2018–2019 and 2019–2020). To determine the date of dormancy release, five shoots (20–30 cm in length and 4–8 mm in diameter, with at least 10 flower buds) were randomly sampled every week, starting in late November until the onset of budbreak at the mid February. The shoots were placed on water-soaked florist foam and maintained in a growth chamber with a photosynthetic photon flux density (PPFD) of 70 µmol m^-2^ s^-1^ provided by cool daylight (6500 K) tubes (Osram L58W/865) under a 12-h light photoperiod at 22 ± 1°C for 8 days (Fadón et al., 2018). To determine differences in bud growth, 10 flower buds were randomly picked and weighed on the first and last day in the growth chamber. The date of dormancy release was established when the weight of the flower buds increased by at least 30% after 8 days in the chamber (Fadón and Rodrigo, 2019).

In peach, the date of dormancy release was measured as Leida et al. (2012). Briefly, 10 budsticks from three different trees were placed in a chamber with a PPFD of 27 µmol m^-2^ s^-1^ provided by a cool daylight (6500 K) tubes (Osram L58W/765 and set at 24°C 12 h:12 h light:dark cycle. Dormancy release was considered when more than 50% of buds showed at least the green tip of the sepals after 14 days.

### Estimation of Chilling Requirements

Temperatures were recorded hourly at a meteorological station located in the experimental orchard over the two seasons. Chilling was quantified according to the three most commonly used temperature models to quantify chilling in fruit trees (Fadón et al., 2020). The Chilling Hours model defined “chilling hour” (CH) as 1 h at or below 7.2°C (Weinberger, 1950). The Utah model weights different ranges of temperatures based on their effect on dormancy fulfilment, proposing the use of “chilling-units” (CU) (Richardson et al., 1974). Finally, the Dynamic Model considered the effects of high and mild temperatures during winter on dormancy release and proposed the use of “chilling portions” (CP) (Fishman et al., 1987). In each model, the chilling requirements were considered as the range of values estimated in the 2 years studied.

### Microscope Preparations

Ten flower buds from European plum sampled were fixed in ethanol 95% acetic acid 3:1 (v/v), and then transferred 24 h later to ethanol 75% at 4°C for conservation. For histochemical examination, flower buds were dehydrated in a tertiary butyl alcohol series (70, 85, 95, and 100% v/v), embedded in paraffin wax, sectioned at 10 mm in a Jung 2045 rotatory microtome (Leica Microsystems), and placed onto glass slides previously coated with Haupt’s adhesive. Prior to staining, the sections were rehydrated (three washes of 5 min in Histoclear II [CellPath], one in Histoclar II:ethanol [1:1, v/v] for 5 min, and one in an ethanol series [100, 70, and 40% v/v] for 2 min) (Fadón and Rodrigo, 2019). The samples were then stained using the potassium iodide-iodine reaction (I_2_KI) for 5 min (Fadón et al., 2018). Preparations were observed under a bright field Leica DM250 microscope (Leica Microsystems). Micrographs were taken with an IDS UI-1490SE digital camera with the IDS Software Suite 4.93.0. (IDS Imaging Development Systems GmbH).

### Identification of *DAM* Genes in European Plum Genome

Peach genomic *PpeDAM1-6* sequences and two adjacent genes at each side were selected to perform a BLASTN (Zhang et al., 2000) analysis, with default values, against the *Prunus domestica* Draft Genome Assembly v1.0 (Zhebentyayeva et al., 2019) available at the Genome Database for Rosaceae (GDR; Jung et al., 2019). Only those scaffolds in which the E-value was zero for all the queries were subjected to a synteny analysis utilizing GDR integrated tool Tripal Synteny Viewer, that used MCScanX (Wang et al., 2012) with default settings and blast files resulting from BLASTP with an E-value cutoff of 1·10^-10^, a maximum alignment of 5 and maximum scores of 5. Results were reproduced using ClicO FS, a web-based service to generate circular plots (Cheong et al., 2015). The coding sequences (CDS) of *PdoDAM1-6* were predicted using the web-server tool Prot2gene (http://genomics.brocku.ca/Prot2gene/), using protein sequences from *PpeDAM1-6* as a guide. RNA-seq data were used to manually curate CDS regions for *PdoDAM1-6* previously predicted. Single reads were downloaded from the National Center for Biotechnology Information (NCBI) BioProject database (accession PRJNA630876), and aligned with the *Prunus domestica* Draft Genome Assembly v1.0 using STAR. Representations of both RNA-seq coverage and CDS was performed by means of the R package: Gviz (Hahne and Ivanek, 2016).

### Gene Structural Analysis of *PdoDAM1-6* Genes

Structural distribution of the previously studied genes was compared with other genomes. For every CDS obtained previously BLASTN with default values was performed against each of the different genomes used for comparison. The best hit for the first and last nucleotide position was taken as the genomic coordinates to draw the diagram. Diagram was generated using the “Gviz” R package for genomic representations mentioned above.

### Phylogenetic Analysis of Plum *DAM* Genes

DAMs and DAM-related protein sequences from other species were downloaded from GeneBank TAIR and PlantTFDB. Their accession numbers and their references are shown in **Supplementary Table S4**. These sequences along the predicted protein sequences of PdoDAM1-6 constituted the input for performing a multiple alignment with Clustal Omega (Sievers et al., 2011). Blocks of highly homologous regions were selected from the multiple alignment file for further analysis using Gblocks (Talavera and Castresana, 2007). Phylogenetic tree was performed using MEGA 7 (Kumar et al., 2016) with Maximum Likelihood method based on Jones-Taylor-Thorton (JTT) matrix-based model (Jones et al., 1992) and allowing for invariable sites (+I) and using a discrete gamma model (+G) (Yang, 1994). The tree was tested using a bootstrap of 1,000 replicates, removing the nodes with less than 70% bootstrap confidence.

### Analysis of Cis-Elements in *PdoDAM1-6* Regulatory Regions

The cis-elements were predicted using the genomic sequences of *PdoDAM1-6* genes from the promoter until the start of the second exon, by means of PlantCARE (http://bioinformatics.psb.ugent.be/webtools/plantcare/html/) (Lescot et al., 2002) and PLACE databases (http://www.dna.affrc.go.jp/PLACE/) (Higo et al., 1999).

### Expression Analysis by Real-Time Quantitative PCR (RT-qPCR)

Total RNA was isolated from bud samples using Plant/Fungi Total RNA Purification Kit (Norgen, Thorold). Lysis buffer was supplemented with Polyvinylpyrrolidone (PVP-40) 1% (w/v) right before usage. Potential genomic DNA was removed with the RNAse-free DNAse I kit (Norgen Thorold). After assessing RNA integrity by gel electrophoresis, 500 ng of each sample were retrotranscribed using PrimeScript RT reagent kit (Takara Bio) in a total volume of 10 μl. Twenty-fold diluted samples were used to perform RT-qPCR; 2 μl in a total volume of 20 μl was analyzed in each well. RT-qPCR was conducted on a StepOnePlus Real-Time PCR System (Life Technologies) using SYBR premix Ex Taq (Tli RNseH plus) (Takara Bio). Steps used for the chain reaction were: an initial incubation of 10 min at 95°C, followed by 40 recurred cycles of 15 s at 95°C and 1 min at 60°C each. Amplification specificity was assessed both by finding a unique peak in the melting curve and by amplicon size estimation using gel electrophoresis. *Actin*-like and *AGL26*-like genes were used as reference genes since they were previously described as suitable for plum expression experiments by Lloret et al. (2017a). The stability of the selected housekeeping genes was analyzed with two different software: BestKeeper (Pfaffl et al., 2004) and geNorm (Vandesompele et al., 2002). BestKeeper recommended that reference gene should have a SD value <1.0 to be considered suitable for normalization and herein both genes had an SD value of 0,40. On the other hand, according to geNorm program, genes with an M-value below the threshold of 1.5 were considered stably expressed and the obtained value was M = 0,424. The geometric mean of Ct values obtained for the reference genes was used to normalize the Ct values obtained for each gene analysis in a sample dependent manner. For every amplification experiment, three independent biological samples with two technical replicates each were analyzed. In addition, a relative standard curve was built in order to obtain relative expression values that were averaged to obtain the final results. In the cases where two possible isoforms were noticed (*PdoDAM4* and *PdoDAM5*), differential primers were designed to determine which form was more abundant during bud development. For each timepoint, RT-qPCR using the two possible combination of primers was performed in parallel, as described above, at the same PCR instrument. Efficiency (E) of each primer pair was determined using a relative standard curve and was used to correct Ct values and allow comparison between them. In every case the abundant isoform was taken as a reference and the minoritarian was calculated as a percentage of it. The primers used to amplify each gene are presented in **Supplementary Table S5**.

### Statistical Analysis

Experimental values were processed using Statgraphics XVI.I package 324 (Statpoint Technologies) to evaluated the statistics significance. Klustal-Wallis test with a confidence level of 95% was used for comparison of multiple samples. Different letters mean significantly different samples.

## Data Availability Statement

The RNA-seq data analyzed for this study can be found in the National Center for Biotechnology Information (NCBI) BioProject database ID PRJNA630876 (http://www.ncbi.nlm.nih.gov/bioproject/630876).

## Author Contributions

CQ-T and AL performed RT-qPCR experiments and bioinformatic analyses. BG and JR performed dormancy measurements and microscopic analysis in European plum. MB, JR, GR, and AL conceived and designed the experiments. GR and AL wrote the paper. All authors contributed to the article and approved the submitted version.

## Funding

This research was funded by Instituto Nacional de Investigación y Tecnología Agraria y Alimentaria (INIA)-FEDER (RFP2015-00015-00, RTA2017-00003-00, RTA2017-00011-C03-01) and the Gobierno de Aragón–European Social Fund, European Union (Grupo Consolidado A12_17R). CQ-T was funded by a fellowship co-financed by the European Social Fund and the Instituto Valenciano de Investigaciones Agrarias (IVIA). BG was supported by a fellowship of Consejo Nacional de Ciencia y Tecnología of México (CONACYT, 471839). AL was funded by a fellowship of Ministerio de Ciencia, Innovación y Universidades of Spanish Government.

## Conflict of Interest

The authors declare that the research was conducted in the absence of any commercial or financial relationships that could be construed as a potential conflict of interest.

## References

[B1] Arora R. Wisniewski M. E. (1994). Cold acclimation in genetically related (sibling) deciduous and evergreen peach (*Prunus persica* [L.] Batsch): II. A 60-kilodalton bark protein in cold-acclimated tissues of peach is heat stable and related to the dehydrin family of proteins. Plant Physiol. 105, 95–101. 10.1104/pp.105.1.95 8029367 PMC159333

[B2] Arora R. Wisniewski M. E. Scorza R. (1992). Cold acclimation in genetically related (sibling) deciduous and evergreen peach (*Prunus persica* [L.] Batsch): I. Seasonal changes in cold hardiness and polypeptides of bark and xylem tissues. Plant Physiol. 99, 1562–1568. 10.1104/pp.99.4.1562 16669074 PMC1080664

[B3] Arora R. Wisniewski M. Rowland L. J. (1996). Cold acclimation and alterations in dehydrin-like and bark storage proteins in the leaves of sibling deciduous and evergreen peach. J. Am. Soc Hortic. Sci. 121, 915–919. 10.21273/JASHS.121.5.915

[B4] Artlip T. S. Callahan A. M. Bassett C. L. Wisniewski M. E. (1997). Seasonal expression of a dehydrin gene in sibling deciduous and evergreen genotypes of peach (*Prunus persica* [L.] Batsch). Plant Mol. Biol. 33, 61–70. 10.1023/a:1005787909506 9037159

[B5] Baggiolini M. (1952). Les stades repérés des arbres fruitiers à noyau. Rev. Romande Agric. Vitic. Arboric. 8, 3–4.

[B6] Balogh E. Halász J. Soltész A. Erös-Honti Z. Gutermuth Á. Szalay L. (2019). Identification, structural and functional characterization of dormancy regulator genes in apricot (*Prunus armeniaca* L.). Front. Plant Sci. 10, 402, 1–16. 10.3389/fpls.2019.00402 31024581 PMC6460505

[B7] Bielenberg D. G. Wang Y. Fan S. Reighard G. L. Scorza R. Abbott A. G. (2004). A deletion affecting several gene candidates is present in the evergrowing peach mutant. J. Hered. 95, 436–444. 10.1093/jhered/esh057 15388771

[B8] Bielenberg D. G. Wang Y. Li Z. Zhebentyayeva T. Fan S. Reighard G. L. (2008). Sequencing and annotation of the evergrowing locus in peach [*Prunus persica* (L.) Batsch] reveals a cluster of six MADS-box transcription factors as candidate genes for regulation of terminal bud formation. Tree Genet. Genomes 4, 495–507. 10.1007/s11295-007-0126-9

[B9] Cheong W.-H. Tan Y.-C. Yap S.-J. Ng K.-P. (2015). ClicO FS: an interactive web-based service of Circos. Bioinform 31, 3685–3687. 10.1093/bioinformatics/btv433 PMC4817113 26227146

[B10] Conde D. Perales M. Sreedasyam A. Tuskan G. A. Lloret A. Badenes M. L. (2019). Engineering tree seasonal cycles of growth through chromatin modification. Front. Plant Sci. 10 (412). 10.3389/fpls.2019.00412 31024588 PMC6459980

[B11] Cooke J. E. K. Eriksson M. E. Junttila O. (2012). The dynamic nature of bud dormancy in trees: environmental control and molecular mechanisms. Plant Cell Environ. 35, 1707–1728. 10.1111/j.1365-3040.2012.02552.x 22670814

[B12] de la Fuente L. Conesa A. Lloret A. Badenes M. L. Ríos G. (2015). Genome-wide changes in histone H3 lysine 27 trimethylation associated with bud dormancy release in peach. Tree Genet. Genomes 11, 45–45. 10.1007/s11295-015-0869-7

[B13] Deng W. Buzas D. M. Ying H. Robertson M. Taylor J. Peacock W. J. (2013). *Arabidopsis* polycomb repressive complex2 binding sites contain putative GAGA factor binding motifs within coding regions of genes. BMC Genomics 14 (593). 10.1186/1471-2164-14-593 24001316 PMC3766684

[B14] Erez A. (2000). “Bud dormancy; phenomenon, problems and solutions in the tropics and subtropics,” in Temperate fruit crops in warm climates. Ed. Erez A. (Dordrecht: Springer Netherlands), 17–48. 10.1007/978-94-017-3215-4_2

[B15] Esmenjaud D. Dirlewanger E. (2007). “Plum,” in Fruits and nuts. Ed. Kole C. (Berlin, Heidelberg: Springer), 119–135. 10.1007/978-3-540-34533-6_4

[B16] Fadón E. Rodrigo J. (2018). Unveiling winter dormancy through empirical experiments. Environ. Exp. Bot. 152, 28–36. 10.1016/j.envexpbot.2017.11.006

[B17] Fadón E. Rodrigo J. (2019). Combining histochemical staining and image analysis to quantify starch in the ovary primordia of sweet cherry during winter dormancy. J. Vis. Exp. 20, 145. 10.3791/58524 30958482

[B18] Fadón E. Herrero M. Rodrigo J. (2015). Flower development in sweet cherry framed in the BBCH scale. Sci. Hortic. 192, 141–147. 10.1016/j.scienta.2015.05.027

[B19] Fadón E. Herrero M. Rodrigo J. (2018). Dormant flower buds actively accumulate starch over winter in sweet cherry. Front. Plant Sci. 9:171:171. 10.3389/fpls.2018.00171 29497434 PMC5818465

[B20] Fadón E. Herrera S. Guerrero B. II Guerra M. E. Rodrigo J. (2020). Chilling and heat requirements of temperate stone fruit trees (*Prunus* sp.). Agron 10 (409). 10.3390/agronomy10030409

[B21] Falavigna V. da S. Porto D. D. Buffon V. Margis-Pinheiro M. Pasquali G. (2014). Differential transcriptional profiles of dormancy-related genes in apple buds. Plant Mol. Biol. Rep. 32, 796–813. 10.1007/s11105-013-0690-0

[B22] Falavigna V. da S. Porto D. D. Miotto Y. E. Santos H. P. D. de Oliveira P. R. D. (2018). Evolutionary diversification of galactinol synthases in Rosaceae: adaptive roles of galactinol and raffinose during apple bud dormancy. J. Exp. Bot. 69, 1247–1259. 10.1093/jxb/erx451 29373762 PMC6018919

[B23] Fishman S. Erez A. Couvillon G. A. (1987). The temperature dependence of dormancy breaking in plants: mathematical analysis of a two-step model involving a cooperative transition. J. Theor. Biol. 124, 473–483. 10.1016/S0022-5193(87)80221-7

[B24] Fu D. Mason A. S. Xiao M. Yan H. (2016). Effects of genome structure variation, homeologous genes and repetitive DNA on polyploid crop research in the age of genomics. Plant Sci. 242, 37–46. 10.1016/j.plantsci.2015.09.017 26566823

[B25] Gharbi O. Wünsch A. Rodrigo J. (2014). Characterization of accessions of ‘Reine Claude Verte’ plum using *Prunus* SRR and phenotypic traits. Sci. Hortic. 169, 57–65. 10.1016/j.scienta.2014.02.018

[B26] Gregis V. Andrés F. Sessa A. Guerra R. F. Simonini S. Mateos J. L. (2013). Identification of pathways directly regulated by *SHORT VEGETATIVE PHASE* during vegetative and reproductive development in Arabidopsis. Genome Biol. 14, R56. 10.1186/gb-2013-14-6-r56 23759218 PMC3706845

[B27] Guan Y.-F. Huang X.-Y. Zhu J. Gao J.-F. Zhang H.-X. Yang Z.-N. (2008). *RUPTURED POLLEN GRAIN1*, a member of the MtN3/saliva gene family, is crucial for exine pattern formation and cell integrity of microspores in Arabidopsis. Plant Physiol. 147, 852–863. 10.1104/pp.108.118026 18434608 PMC2409014

[B28] Guo L. Dai J. Ranjitkar S. Yu H. Xu J. Luedeling E. (2014). Chilling and heat requirements for flowering in temperate fruit trees. Int. J. Biometeorol. 58, 1195–1206. 10.1007/s00484-013-0714-3 23958788

[B29] Haake V. Cook D. Riechmann J. L. Pineda O. Thomashow M. F. Zhang J. Z. (2002). Transcription factor CBF4 is a regulator of drought adaptation in Arabidopsis. Plant Physiol. 130, 639–648. 10.1104/pp.006478 12376631 PMC166593

[B30] Hahne F. Ivanek R. (2016). Visualizing genomic data using gviz and bioconductor. Methods Mol. Biol. 1418, 335–351. 10.1007/978-1-4939-3578-9_16 27008022

[B31] Hänninen H. Tanino K. (2011). Tree seasonality in a warming climate. Trends Plant Sci. 16, 412–416. 10.1016/j.tplants.2011.05.001 21640632

[B32] Hao X. Chao W. Yang Y. Horvath D. (2015). Coordinated expression of *FLOWERING LOCUS T* and *DORMANCY ASSOCIATED MADS-BOX*-like genes in leafy spurge. PloS One 10, e0126030. 10.1371/journal.pone.0126030 25961298 PMC4427404

[B33] Heide O. M. Prestrud A. K. (2005). Low temperature, but not photoperiod, controls growth cessation and dormancy induction and release in apple and pear. Tree Physiol. 25, 109–114. 10.1093/treephys/25.1.109 15519992

[B34] Heide O. M. (2008). Interaction of photoperiod and temperature in the control of growth and dormancy of *Prunus* species. Sci. Hortic. 115, 309–314. 10.1016/j.scienta.2007.10.005

[B35] Higo K. Ugawa Y. Iwamoto M. Korenaga T. (1999). Plant cis-acting regulatory DNA elements (PLACE) database: 1999. Nucleic Acids Res. 27, 297–300. 10.1093/nar/27.1.297 9847208 PMC148163

[B36] Horvath D. P. Chao W. S. Suttle J. C. Thimmapuram J. Anderson J. V. (2008). Transcriptome analysis identifies novel responses and potential regulatory genes involved in seasonal dormancy transitions of leafy spurge (*Euphorbia esula* L.). BMC Genomics 9:536. 10.1186/1471-2164-9-536 19014493 PMC2605480

[B37] Horvath D. P. Sung S. Kim D. Chao W. Anderson J. (2010). Characterization, expression and function of DORMANCY ASSOCIATED MADS-BOX genes from leafy spurge. Plant Mol. Biol. 73, 169–179. 10.1007/s11103-009-9596-5 20066557

[B38] Ibáñez C. Collada C. Casado R. González-Melendi P. Aragoncillo C. Allona I. (2013). Winter induction of the galactinol synthase gene is associated with endodormancy in chestnut trees. Trees 27, 1309–1316. 10.1007/s00468-013-0879-8

[B39] Jacobowitz J. R. Doyle W. C. Weng J.-K. (2019). PRX9 and PRX40 are extensin peroxidases essential for maintaining tapetum and microspore cell wall integrity during Arabidopsis anther development. Plant Cell 31, 848–861. 10.1105/tpc.18.00907 30886127 PMC6501601

[B40] Jiménez S. Lawton-Rauh A. L. Reighard G. L. Abbott A. G. Bielenberg D. G. (2009). Phylogenetic analysis and molecular evolution of the dormancy associated MADS-box genes from peach. BMC Plant Biol. 9:81. 10.1186/1471-2229-9-81 19558704 PMC2713236

[B41] Jiménez S. Reighard G. L. Bielenberg D. G. (2010). Gene expression of *DAM5* and *DAM6* is suppressed by chilling temperatures and inversely correlated with bud break rate. Plant Mol. Biol. 73, 157–167. 10.1007/s11103-010-9608-5 20143130

[B42] Jones D. T. Taylor W. R. Thornton J. M. (1992). The rapid generation of mutation data matrices from protein sequences. Comput. Appl. Biosci. 8, 275–282. 10.1093/bioinformatics/8.3.275 1633570

[B43] Julian C. Rodrigo J. Herrero M. (2011). Stamen development and winter dormancy in apricot (*Prunus armeniaca*). Ann. Bot. 108, 617–625. 10.1093/aob/mcr056 21474504 PMC3170150

[B44] Jung S. Lee T. Cheng C.-H. Buble K. Zheng P. Yu J. (2019). 15 years of GDR: new data and functionality in the Genome Database for Rosaceae. Nucleic Acids Res. 47, D1137–D1145. 10.1093/nar/gky1000 30357347 PMC6324069

[B45] Kumar S. Stecher G. Tamura K. (2016). MEGA7: Molecular evolutionary genetics analysis version 7.0 for bigger datasets. Mol. Biol. Evol. 33, 1870–1874. 10.1093/molbev/msw054 27004904 PMC8210823

[B46] Lang G. A. Early J. D. Martin G. C. Darnell R. L. (1987). Endodormancy, paradormancy, and ecodormancy - physiological terminology and classification for dormancy research. HortScience 22, 371–377.

[B47] Lawton-Rauh A. (2003). Evolutionary dynamics of duplicated genes in plants. Mol. Phylogenet. Evol. 29, 396–409. 10.1016/j.ympev.2003.07.004 14615182

[B48] Legave J.-M. Guédon Y. Malagi G. El Yaacoubi A. Bonhomme M. (2015). Differentiated responses of apple tree floral phenology to global warming in contrasting climatic regions. Front. Plant Sci. 6 (1054), 1–13. 10.3389/fpls.2015.01054 26697028 PMC4678210

[B49] Leida C. Terol J. Martí G. Agustí M. Llácer G. Badenes M. L. (2010). Identification of genes associated with bud dormancy release in *Prunus persica* by suppression subtractive hybridization. Tree Physiol. 30, 655–666. 10.1093/treephys/tpq008 20231169

[B50] Leida C. Conesa A. Llácer G. Badenes M. L. Ríos G. (2012). Histone modifications and expression of *DAM6* gene in peach are modulated during bud dormancy release in a cultivar-dependent manner. New Phytol. 193, 67–80. 10.1111/j.1469-8137.2011.03863.x 21899556

[B51] Leitch A. R. Leitch I. J. (2008). Genomic plasticity and the diversity of polyploid plants. Science 320, 481–483. 10.1126/science.1153585 18436776

[B52] Lescot M. Déhais P. Thijs G. Marchal K. Moreau Y. Van de Peer Y. (2002). PlantCARE, a database of plant cis-acting regulatory elements and a portal to tools for in silico analysis of promoter sequences. Nucleic Acids Res. 30, 325–327. 10.1093/nar/30.1.325 11752327 PMC99092

[B53] Li Z. Reighard G. L. Abbott A. G. Bielenberg D. G. (2009). Dormancy-associated MADS genes from the *EVG* locus of peach [*Prunus persica* (L.) Batsch] have distinct seasonal and photoperiodic expression patterns. J. Exp. Bot. 60, 3521–3530. 10.1093/jxb/erp195 19553369 PMC2724702

[B54] Liu J. Sherif S. M. (2019). Hormonal orchestration of bud dormancy cycle in deciduous woody perennials. Front. Plant Sci. 10 (1136). 10.3389/fpls.2019.01136 31620159 PMC6759871

[B55] Lloret A. Conejero A. Leida C. Petri C. Gil-Muñoz F. Burgos L. (2017a). Dual regulation of water retention and cell growth by a stress-associated protein (SAP) gene in *Prunus*. Sci. Rep. 7, 332. 10.1038/s41598-017-00471-7 28336950 PMC5428470

[B56] Lloret A. Martínez-Fuentes A. Agustí M. Badenes M. L. Ríos G. (2017b). Chromatin-associated regulation of sorbitol synthesis in flower buds of peach. Plant Mol. Biol. 95, 507–517. 10.1007/s11103-017-0669-6 29038917

[B57] Lloret A. Badenes M. L. Ríos G. (2018). Modulation of dormancy and growth responses in reproductive buds of temperate trees. Front. Plant Sci. 9 (1368), 1–12. 10.3389/fpls.2018.01368 30271422 PMC6146825

[B58] Ludevid D. Höfte H. Himelblau E. Chrispeels M. J. (1992). The expression pattern of the tonoplast intrinsic protein gamma-TIP in *Arabidopsis thaliana* is correlated with cell enlargement. Plant Physiol. 100, 1633–1639. 10.1104/pp.100.4.1633 16653178 PMC1075845

[B59] Luedeling E. Girvetz E. H. Semenov M. A. Brown P. H. (2011). Climate change affects winter chill for temperate fruit and nut trees. PloS One 6, e20155. 10.1371/journal.pone.0020155 21629649 PMC3101230

[B60] Maurel C. Boursiac Y. Luu D.-T. Santoni V. Shahzad Z. Verdoucq L. (2015). Aquaporins in plants. Physiol. Rev. 95, 1321–1358. 10.1152/physrev.00008.2015 26336033

[B61] Mimida N. Saito T. Moriguchi T. Suzuki A. Komori S. Wada M. (2015). Expression of *DORMANCY-ASSOCIATED MADS-BOX (DAM)*-like genes in apple. Biol. Plant 59, 237–244. 10.1007/s10535-015-0503-4

[B62] Niu Q. Li J. Cai D. Qian M. Jia H. Bai S. (2016). Dormancy-associated MADS-box genes and microRNAs jointly control dormancy transition in pear (*Pyrus pyrifolia* white pear group) flower bud. J. Exp. Bot. 67, 239–257. 10.1093/jxb/erv454 26466664 PMC4682432

[B63] Petri C. Burgos L. (2005). Transformation of fruit trees. Useful breeding tool or continued future prospect? Transgenic Res. 14, 15–26. 10.1007/s11248-004-2770-2 15865045

[B64] Petri C. Alburquerque N. Faize M. Scorza R. Dardick C. (2018). Current achievements and future directions in genetic engineering of European plum (*Prunus domestica* L.). Transgenic Res. 27, 225–240. 10.1007/s11248-018-0072-3 29651659 PMC5986827

[B65] Pfaffl M. W. Tichopad A. Prgomet C. Neuvians T. P. (2004). Determination of stable housekeeping genes, differentially regulated target genes and sample integrity: bestKeeper-excel-based tool using pair-wise correlations. Biotechnol. Lett. 26 (6), 509–515. 10.1023/b:bile.0000019559.84305.47 15127793

[B66] Prudencio Á.S. Werner O. Martínez-García P. J. Dicenta F. Ros R. M. Martínez-Gómez P. (2018). DNA methylation analysis of dormancy release in almond (*Prunus dulcis*) Flower buds using epi-genotyping by sequencing. Int. J. Mol. Sci. 19 (11), 3542 1–18. 10.3390/ijms19113542 PMC6274898 30423798

[B67] Richardson E. A. Seeley S. D. Walker D. R. Seeley S. D. Walker D. II (1974). A model for estimating the completion of rest for “Redhaven” and “Elberta” peach trees. HortScience 9, 331–332.

[B68] Rinne P. L. Kaikuranta P. M. van der Schoot C. (2001). The shoot apical meristem restores its symplasmic organization during chilling-induced release from dormancy. Plant J. 26, 249–264. 10.1046/j.1365-313x.2001.01022.x 11439114

[B69] Rinne P. L. H. Welling A. Vahala J. Ripel L. Ruonala R. Kangasjärvi J. (2011). Chilling of dormant buds hyperinduces FLOWERING LOCUS T and recruits GA-inducible 1,3-beta-glucanases to reopen signal conduits and release dormancy in Populus. Plant Cell 23, 130–146. 10.1105/tpc.110.081307 21282527 PMC3051240

[B70] Ríos G. Tadeo F. R. Leida C. Badenes M. L. (2013). Prediction of components of the sporopollenin synthesis pathway in peach by genomic and expression analyses. BMC Genomics 14 (40). 10.1186/1471-2164-14-40 23331975 PMC3556096

[B71] Ríos G. Leida C. Conejero A. Badenes M. L. (2014). Epigenetic regulation of bud dormancy events in perennial plants. Front. Plant Sci. 5:247:247. 10.3389/fpls.2014.00247 24917873 PMC4042555

[B72] Rose A. B. (2019). Introns as gene regulators: a brick on the accelerator. Front. Genet. 9 (672), 1–6. 10.3389/fgene.2018.00672 30792737 PMC6374622

[B73] Rothkegel K. Sánchez E. Montes C. Greve M. Tapia S. Bravo S. (2017). DNA methylation and small interference RNAs participate in the regulation of MADS-box genes involved in dormancy in sweet cherry (*Prunus avium* L.). Tree Physiol. 37, 1739–1751. 10.1093/treephys/tpx055 28541567

[B74] Saito T. Bai S. Imai T. Ito A. Nakajima I. Moriguchi T. (2015). Histone modification and signalling cascade of the dormancy-associated MADS-box gene, *PpMADS13-1*, in Japanese pear (*Pyrus pyrifolia*) during endodormancy. Plant Cell Environ. 38, 1157–1166. 10.1111/pce.12469 25311427

[B75] Sasaki R. Yamane H. Ooka T. Jotatsu H. Kitamura Y. Akagi T. (2011). Functional and expressional analyses of *PmDAM* genes associated with endodormancy in Japanese apricot. Plant Physiol. 157, 485–497. 10.1104/pp.111.181982 21795580 PMC3165894

[B76] Sievers F. Wilm A. Dineen D. Gibson T. J. Karplus K. Li W. (2011). Fast, scalable generation of high-quality protein multiple sequence alignments using Clustal Omega. Mol. Syst. Biol. 7, 539. 10.1038/msb.2011.75 21988835 PMC3261699

[B77] Singh R. K. Maurya J. P. Azeez A. Miskolczi P. Tylewicz S. Stojkovič K. (2018). A genetic network mediating the control of bud break in hybrid aspen. Nat. Commun. 9, 4173. 10.1038/s41467-018-06696-y 30301891 PMC6177393

[B78] Tabuenca M. C. Iturrioz M. (1991). Description of European plum varieties, 2: Green Queen Claudia. Estac. Exp. Aula Dei. 20.

[B79] Tabuenca M. C. (1967). Winter chilling requirements of plum varieties. Estac. Exp. Aula Dei. 8, 383–391.

[B80] Talavera G. Castresana J. (2007). Improvement of phylogenies after removing divergent and ambiguously aligned blocks from protein sequence alignments. Syst. Biol. 56, 564–577. 10.1080/10635150701472164 17654362

[B81] Tuan P. A. Bai S. Saito T. Ito A. Moriguchi T. (2017). *Dormancy-Associated MADS-Box (DAM)* and the abscisic acid pathway regulate pear endodormancy through a feedback mechanism. Plant Cell Physiol. 58, 1378–1390. 10.1093/pcp/pcx074 28586469

[B82] Tylewicz S. Petterle A. Marttila S. Miskolczi P. Azeez A. Singh R. K. (2018). Photoperiodic control of seasonal growth is mediated by ABA acting on cell-cell communication. Science 360, 212–215. 10.1126/science.aan8576 29519919

[B83] Vandesompele J. De Preters K. Pattyn F. Poppe B. Van Roy N. De Paepe A. (2002). Accurate normalization of real-time quantitative RT-PCR data by geometric averaging of multiple internal control genes. Genome Biol. 3 (7), research0034.1. 10.1186/gb-2002-3-7-research0034 12184808 PMC126239

[B84] Vimont N. Quah F. X. Schoepfer D. G. Roudier F. Dirlewanger E. Wigge P. A. (2020). ChIP-seq and RNA-seq for complex and low-abundance tree buds reveal chromatin and expression co-dynamics during sweet cherry bud dormancy. Tree Genet. Genomes 16 (9). 10.1007/s11295-019-1395-9

[B85] Vitasse Y. Signarbieux C. Fu Y. H. (2018). Global warming leads to more uniform spring phenology across elevations. Proc. Natl. Acad. Sci. U. S. A. 15 (5), 1004–1008. 10.1073/pnas.1717342115 PMC5798366 29279381

[B86] Wang Y. Tang H. Debarry J. D. Tan X. Li J. Wang X. (2012). MCScanX: a toolkit for detection and evolutionary analysis of gene synteny and collinearity. Nucleic Acids Res. 40, e49. 10.1093/nar/gkr1293 22217600 PMC3326336

[B87] Wang Q. Xu G. Zhao X. Zhang Z. Wang X. Liu X. (2020). Transcription factor TCP20 regulates peach bud endodormancy by inhibiting *DAM5/DAM6* and interacting with ABF2. J. Exp. Bot. 71, 1585–1597. 10.1093/jxb/erz516 31740930 PMC7031059

[B88] Weinberger J. H. (1950). Chilling requirements of peach varieties. P. Am. Soc. Hortic. Sci. 56, 122–128.

[B89] Welling A. Palva E. T. (2006). Molecular control of cold acclimation in trees. Physiol. Plant 127, 167–181. 10.1111/j.1399-3054.2006.00672.x

[B90] Wells C. E. Vendramin E. Jimenez Tarodo S. Verde I. Bielenberg D. G. (2015). A genome-wide analysis of MADS-box genes in peach [*Prunus persica* (L.) Batsch]. BMC Plant Biol. 15 (41), 1–16. 10.1186/s12870-015-0436-2 25848674 PMC4329201

[B91] Wisniewski M. Norelli J. Bassett C. Artlip T. Macarisin D. (2011). Ectopic expression of a novel peach (Prunus persica) CBF transcription factor in apple (Malus × domestica) results in short-day induced dormancy and increased cold hardiness. Planta 233, 971–983. 10.1007/s00425-011-1358-3 21274560

[B92] Wisniewski M. Norelli J. Artlip T. (2015). Overexpression of a peach CBF gene in apple: a model for understanding the integration of growth, dormancy, and cold hardiness in woody plants. Front. Plant Sci. 6 (85), 1–13. 10.3389/fpls.2015.00085 25774159 PMC4343015

[B93] Wisniewski M. Nassuth A. Arora R. (2018). Cold hardiness in trees: a mini-review. Front. Plant Sci. 9 (1394), 1–9. 10.3389/fpls.2018.01394 30294340 PMC6158558

[B94] Woznicki T. L. Heide O. M. Sønsteby A. Måge F. Remberg S. F. (2019). Climate warming enhances flower formation, earliness of blooming and fruit size in plum (*Prunus domestica* L.) in the cool Nordic environment. Sci. Hortic. 257 (108750). 10.1016/j.scienta.2019.108750

[B95] Wu R.-M. Walton E. F. Richardson A. C. Wood M. Hellens R. P. Varkonyi-Gasic E. (2012). Conservation and divergence of four kiwifruit SVP-like MADS-box genes suggest distinct roles in kiwifruit bud dormancy and flowering. J. Exp. Bot. 63, 797–807. 10.1093/jxb/err304 22071267 PMC3254681

[B96] Wu R. Tomes S. Karunairetnam S. Tustin S. D. Hellens R. P. Allan A. C. (2017). *SVP*-like MADS Box genes control dormancy and budbreak in apple. Front. Plant Sci. 8 (477). 10.3389/fpls.2017.00477 28421103 PMC5378812

[B97] Wu R. Wang T. Richardson A. C. Allan A. C. Macknight R. C. Varkonyi-Gasic E. (2019). Histone modification and activation by SOC1-like and drought stress-related transcription factors may regulate AcSVP2 expression during kiwifruit winter dormancy. Plant Sci. 281, 242–250. 10.1016/j.plantsci.2018.12.001 30824057

[B98] Yamane H. Wada M. Honda C. Matsuura T. Ikeda Y. Hirayama T. (2019). Overexpression of *Prunus DAM6* inhibits growth, represses bud break competency of dormant buds and delays bud outgrowth in apple plants. PloS One 14, e0214788. 10.1371/journal.pone.0214788 30964897 PMC6456227

[B99] Yang Q. Yang B. Li J. Wang Y. Tao R. Yang F. (2020). ABA-responsive ABRE-BINDING FACTOR3 activates *DAM3* expression to promote bud dormancy in Asian pear. Plant Cell Environ. 43, 1360–1375. 10.1111/pce.13744 32092154

[B100] Yang Z. (1994). Maximum likelihood phylogenetic estimation from DNA sequences with variable rates over sites: approximate methods. J. Mol. Evol. 39, 306–314. 10.1007/bf00160154 7932792

[B101] Yu J. Conrad A. O. Decroocq V. Zhebentyayeva T. Williams D. E. Bennett D. (2020). Distinctive gene expression patterns define endodormancy to ecodormancy transition in apricot and peach. Front. Plant Sci. 11 (180), 1–24. 10.3389/fpls.2020.00180 32180783 PMC7059448

[B102] Zhang Z. Schwartz S. Wagner L. Miller W. (2000). A greedy algorithm for aligning DNA sequences. J. Comput. Biol. 7, 203–214. 10.1089/10665270050081478 10890397

[B103] Zhang Q. Chen W. Sun L. Zhao F. Huang B. Yang W. (2012). The genome of *Prunus mume*. Nat. Commun. 3, 1–8. 10.1038/ncomms2290 PMC3535359 23271652

[B104] Zhao K. Zhou Y. Ahmad S. Xu Z. Li Y. Yang W. (2018a). Comprehensive cloning of *Prunus mume* dormancy associated MADS-box genes and their response in flower bud development and dormancy. Front. Plant Sci. 9 (17). 10.3389/fpls.2018.00017 29449849 PMC5800298

[B105] Zhao K. Zhou Y. Ahmad S. Yong X. Xie X. Han Y. (2018b). *PmCBFs* synthetically affect *PmDAM6* by alternative promoter binding and protein complexes towards the dormancy of bud for *Prunus mume*. Sci. Rep. 8, 1–10. 10.1038/s41598-018-22537-w 29540742 PMC5852209

[B106] Zhebentyayeva T. Shankar V. Scorza R. Callahan A. Ravelonandro M. Castro S. (2019). Genetic characterization of worldwide *Prunus domestica* (plum) germplasm using sequence-based genotyping. Hortic. Res. 6, 1–13. 10.1038/s41438-018-0090-6 30603097 PMC6312543

[B107] Zhu C. Perry S. E. (2005). Control of expression and autoregulation of *AGL15*, a member of the MADS-box family. Plant J. 41, 583–594. 10.1111/j.1365-313X.2004.02320.x 15686521

[B108] Zhu Y. Li Y. Xin D. Chen W. Shao X. Wang Y. (2015). RNA-Seq-based transcriptome analysis of dormant flower buds of Chinese cherry (*Prunus pseudocerasus*). Gene 555, 362–376. 10.1016/j.gene.2014.11.032 25447903

