# Supplementary material for: Structure and Expression of Bud Dormancy-Associated MADS-Box Genes (DAM) in European Plum
Source: Front Plant Sci. 2020 Aug 19;11:1288. doi: 10.3389/fpls.2020.01288 (PMC7466548; doi:10.3389/fpls.2020.01288)
Supplement: Supplementary file 1 [file Table_1.docx]

Supplementary Material

# Supplementary Figures and Tables

## Supplementary Figures

2018-2019

2019-2020

Oct Nov Dec Jan Feb

0

20

40

60

80

100

Chilling portions

**Supplementary Figure S1.** Winter chilling accumulation (chilling portions) in Montañana, Zaragoza (Spain) during two years. Snowflakes: dates of chilling fulfilment for European plum cv. ‘Reine Claude Verte’.

>PdoDAM1

ATGATGAGGGAGAAGATCAAGATCAAGAAGATTGACAACTTGCCTGCAAGGCAAGTGACCTTCTCAAAGAGGAGGAGAGGGATCTTCAAGAAAGCTGCAGAGTTATCTGTTCTGTGTGAATCTGAGGTGGCAGTTGTCATCTTTTCTGCTACTGGCAAGCTATTTGATTATTCAAGCTCAAGTATGAAGGATGTTATTGAAAGGTACCAAGCGCACATAAATGGTGGTGAAAAATTTGACGAACCGTCTATTGAGTTGCAGCCAGAGAATGAAAACCACATCAGATTGAGCAAGGAACTTGAGGAGAAGAGCCGCCAGCTGAGGCAGATGAAAGGAGAGGATCTTGAAGAGCTGAATTTTGATGAGTTGCAGAAGTTAGAACAACTGGTGGATGCAAGCCTTGGCCGTGTGATTGAAACTAAGGATGAACGGATTATGAGTGAGATTATGGCACTTGAAAGAAAGAGAGCTGAGCTTGTAAAAGCCAACAAACAGCTAAGGCAGAGGCAAATGTTATCCAGAGGAAATATTGGACCTGCGCTTATGGAGCCGGAGAGGTTGAATAATAATATTGGTGGTGGAGGAGAAGAAGAAGGCATGTCATCTGAATCTGCTACCTCCACCACCTGCAACAGTGCTCCCAGTCTCTCTCTTGAAGATGACTCCGACGACGTCACTTTATCTCTCAAACTGGGGCTTCCATAA

>PdoDAM2

ATGGTGAAGGCGATGAGGAAGAAGATCAAGATCAAGAAGATTGACTACTTGCCTGCAAGGCAGGTGACCTTCTCAAAGAGGAGGAGAGGGATCTTCAAGAAAGCTGAGGAGCTATCTGTTCTGTGTGAATCTGAGGTGGCAGTTGTCATCTTTTCTGCTACTGGCAAGCTTTTTGATTATTCAAGCTCAAGTACAAAGGATGTTGTTGAAAGGTATAAAGCACACACAAATGGTGTCGAAAAATCAGACGAACCGTCTGTTGAGCTGCAGCTAGAAATTGAAAACCACATCAGATTGACCAAGGAACTTGAGGAGAAGAGCCGCCAGCTGAGGCAGATGAGAGGAGAGGATCTTGAAGAGCTGAATATTGATGAGTTGCAGAAGTTAGAACAACTGGTGGATGCAAGCCTTGGCCGTGTGATTGAAACTAAGGAAGAATTGATTATGAGTGAGATTATGGCACTTGAAAAAAAGGGAGCTGAGCTGGTAGAAGCCAACAACCAGCTACGGCAGAGGATGGTGATGTTATCCAGAGGAAATATTGGACCTGCGCTTACGGAGCCGGAAAGGTTGATTAATAATATTGGAGGTGGAGGAGAAGAAGGCATGTCATCTGAATCTGCCACAAATGCAACCATCAGCAGCTGCAGCAGTGGTCTCAGTCTCTCTCTTGAAGATGACTGCTCCGACGTCACTTTAGCTCTCAAACTGGGGCTTCCCTAA

>PdoDAM3

ATGATGAGGAAGAAGATCAAGATCAAGAAGATTGATTGCTTGCCTGCAAGGCAGGTGACCTTCTCAAAGAGGAGAAGAGGGATCTTGAAGAAAGCTGCCGAGCTATCTGTTCTGTGTGAATCTAAGGTGGCAGTTGTCATATTTTCTGCTACCGGCAAGCTTTTTGATTATTCAAGTTCAAGTATCAAGGATGTTATTGAAAGCTACAAAGCGCACAAAAATGGTGTCGAAAAATCGGACGAACCGTCTGTTGAGCTACAGCTAGAGAATGAAAATCACATCGGATTGAGCAAGGAACTCGAGGAGAAGAGCCATCAGCTGAGGCAGATGAAAGCAGAAGATCTTGAAGAGCTGGATTTTGATGAGTTGCAGAAGTTAGAACAACTGGTGGACGCAAGCCTTAGCCGTGTGATTGAAACTAAGGAAGAACTGAGAATGACAGAGATTACGGCACTTGAAAGAAAGGGAGCTGAGCTGGTAGAAGCCAACAACCAGCTAAGGCAGACGATGGTGATGTTATCCGGAGGACATACTGGACCTACGCTTAAGGATCCTGAGAGCTTGAGTGATAAGACCGGAGGTGGAGGAGAAGAAGAAGGCATGTCATCTGAATCTGCTATCTCCACCACCTGCAACAGTGCTCTCAGTCTCTCCCTTGGAGATGACTCCGACGACGTCACATTATCTCTCAAACTGGGGCTTCCTTAA

>PdoDAM4.1

ATGGTGAAAATGATGAGGGAGAAGATCAAGATCAAGAAGATTGACTACCTGCCAGCAAGGCAGGTGACCTTCTCAAAGAGGAGAAGAGGGATATTCAAGAAAGCTGCAGAGCTATCTGTTCTGTGTGAATCTGAGGTGGCAGTTGTCATCTTTTCTGCTACTGGCAAGCTTTTTGATTTTTCAAGCTCAAGGATCAAGGATGTTATTGAAAGGTACAAAATGCGCACAAATGGTGTTGAAAAATCGGATGAAGAGTCCCTTGAGCTGCAGCTGGAGAATGAAAACCGCATCAAACTCAGTAAGGAACTCGAGGAGAAGAACCGCCAGCTGAGGCAGATGAAAGGTGAGGATCTTGGATTGCTGGATCTGGATGAGTTGCTGAAGTTGGAACAACTGGTGGAAGCAACCCTTGTCCGTGTGATGGAAACTAAGGAAGAACTGATTATGAGTGATATTGTGGCTCTTGAGAAAAAGGGAACTGAGCTGGTAGAAGCCAACAATCAGATGGCGATGTTAAGGGAGAGGATGGTGATGCTATCCAAAAGAAATACCGGACCTGCCCTTGTGGAGCCATCTGAATCTGCTACCTCCACCAGCTGCAACAGTGCTCTGAGTCTGTCTCTTGAAGATGACTGCTCCGACGACGTCATTTTATCTCTCAAACTGGGGCTAACCGGTTCGCGCGGGTCGTAG

>PdoDAM4.2

ATGGTGAAAATGATGAGGGAGAAGATCAAGATCAAGAAGATTGACTACCTGCCAGCAAGGCAGGTGACCTTCTCAAAGAGGAGAAGAGGGATATTCAAGAAAGCTGCAGAGCTATCTGTTCTGTGTGAATCTGAGGTGGCAGTTGTCATCTTTTCTGCTACTGGCAAGCTTTTTGATTTTTCAAGCTCAAGGATCAAGGATGTTATTGAAAGGTACAAAATGCGCACAAATGGTGTTGAAAAATCGGATGAAGAGTCCCTTGAGCTGCAGCTGGAGAATGAAAACCGCATCAAACTCAGTAAGGAACTCGAGGAGAAGAACCGCCAGCTGAGGCAGATGAAAGGTGAGGATCTTGGATTGCTGGATCTGGATGAGTTGCTGAAGTTGGAACAACTGGTGGAAGCAACCCTTGTCCGTGTGATGGAAACTAAGGAAGAACTGATTATGAGTGATATTGTGGCTCTTGAGAAAAAGGGAACTGAGCTGGTAGAAGCCAACAATCAGATGGCGATGTTAAGGGAGAGGATGGTGATGCTATCCAAAAGAAATACCGGACCTGCCCTTGTGGAGCCATCTGAATCTGCTACCTCCACCAGCTGCAACAGTGCTCTGAGTCTGTCTCTTGAAGATGACTGCTCCGACGACGTCATTTTATCTCTCAAACTGGGGCGTCCGTAA

>PdoDAM5.1

ATGATGAGGAATAAGATCAAGATCAAGAAGATTGACTACTTGCCTGCAAGGCAGGTGACCTTCTCAAAAAGGAGAAGAGGGCTCTTAAAGAAAGCTGCAGAGCTATCTGTTCTCTGTGAATCTGAGGTGGCAGTTGTCATCTTTTCTGCTACTGGCAAGCTTTTTCATTATTCAAGCTCAAGTACCAAGGATGTTATTGAAAGGTACAACGCAGACATGAATGGTGTCGAAAAATCGAACAATCAAGTGATTGAGCTGCAGCTGGAGAATGAAAACCACATCAAACTGAGTAAGGAACTCGAGGAGAAGAGCCGCCAGCTGAGGCAGATGAAAGGTGAGGATCTTCAAAGGCTGAATCTGGATGAGTTGCTGAAGTTGGAACAACTGGTGGAAGCAAGCCTTGGCCGTGTCATGGAAACTAAGGAAGAGCTGATTAAGACTGAGATTATGGAACTTGAAAGAAAGGGAGCTGAGCTAGTTGAAGCCAACAACCAGCTAAGGCAGACGATGGTGATGTTATCTAGAGGAAATACTGGACCTGCGCTTATGGATCAGGAGAGGTTGAATAATAATATTGAAGGTGGAGGAGAAGAAGAAGGCATGTCAGCTGAATCTGCTATCTCCACCACCTGCAACAGTGCTGTCAGTCTCTCTCTTGAAGATGACTCCTCCGATGAGGTCACTTTGTCTCTCAAACTGGGGCGTTAA

>PdoDAM5.2

ATGATGAGGAATAAGATCAAGATCAAGAAGATTGACTACTTGCCTGCAAGGCAGGTGACCTTCTCAAAAAGGAGAAGAGGGCTCTTAAAGAAAGCTGCAGAGCTATCTGTTCTCTGTGAATCTGAGGTGGCAGTTGTCATCTTTTCTGCTACTGGCAAGCTTTTTCATTATTCAAGCTCAAGTACCAAGGATGTTATTGAAAGGTACAACGCAGACATGAATGGTGTCGAAAAATCGAACAATCAAGTGATTGAGCTGCAGCTGGAGAATGAAAACCACATCAAACTGAGTAAGGAACTCGAGGAGAAGAGCCGCCAGCTGAGGCAGATGAAAGGTGAGGATCTTCAAAGGCTGAATCTGGATGAGTTGCTGAAGTTGGAACAACTGGTGGAAGCAAGCCTTGGCCGTGTCATGGAAACTAAGGAAGAGCTGATTAAGACTGAGATTATGGAACTTGAAAGAAAGGGAGCTGAGCTAGTTGAAGCCAACAACCAGCTAAGGCAGACGATGGTGATGTTATCTAGAGGAAATACTGGACCTGCGCTTATGGATCAGGAGAGGTTGAATAATAATATTGAAGGTGGAGGAGAAGAAGAAGGCATGTCAGCTGAATCTGCTATCTCCACCACCTGCAACAGTGCTGTCAGTCTCTCTCTTGAAGATGACTCCTCCGATGAGGTCACTTTGTCTCTCAAACTGGGAAAGCTTCAGTTGAGGAATCCCAACACTGAAAGGGGCTGA

>PdoDAM6

ATGATGAGGGAGAAGATCAAGATCAAGAAGATTGACTACCTGCCAGCAAGGCAGGTTACCTTTTCAAAGAGAAGAAGAGGGCTCTTTAATAAAGCTGCAGAGCTATCTGTTCTGTGTGAATCTGAGGTGGCTGTCGTCATCTTTTCTGCTACTGACAAGCTTTTTGATTATTCAAGCTCAAGTACCAAGGATGTTATTGAAAGGTACAAAGCGCACACAGGTGGTGTTGAAAAATCGGACAAACAGTTTCTTGAGCTGCAACTGGAGAATGAAAACCACATCAAACTGAGTAAGGAAGTCGAGGAGAAGAGCCGCCAGCTGAGGCAGATGAAAGGTGAGGATCTTAAAGGGCTGAATCTCGACGAGCTGCTGAAATTAGAACAACTGCTGGAAGCAAGCCTTGGCCGTGTGATAGAAACTAAGGAAGAGCTGATTATGAGTGAGATTATGGCACTGGAGAAAAAGGGAGCTGAGCTGGCAGAAGCCAACAACCAGTTGAGGCAGAAGATGCAAATGTTATCCGGAGGAAATACTGGACCTGCGTTTGTGGAGCCGGAGACGTTGATTACTAATGTTGGAGGTGGGGGAGAAGAAGACGGCATGTCATCTGAATCTGCCCTAATTGCCACCTCCACCAGCTGCAACAGTGCTGTCAGTCTCTCTCTTGAAGATGACTGCTCCGATGTCACTTTATCTCTCAAACTGGGGCTTCCCTAG

**Supplementary Figure S2.** Sequences of *PdoDAM1-6* genes

## Supplementary Tables

**Supplementary Table S1.** Date of endodormancy breaking and chilling requirements for European plum cv. ‘Reine Claude Verte’ during two years.

| **Season** | **Endodormancy breaking date** | **Chilling Hours** | **Chilling Units** | **Chilling Portions** |
| --- | --- | --- | --- | --- |
| 2018-2019 | 25-Jan | 979 | 1287 | 62.8 |
| 2019-2020 | 27-Jan | 1086 | 1248 | 62.8 |

**Supplementary Table S2.** Minority transcripts of *PdoDAM4* and *PdoDAM5* as a percentage of the more abundant form.

| **Sample name** | **% of DAM4.1 over DAM4.2** | **% of DAM5.2 over DAM5.1** |
| --- | --- | --- |
| **CV1** | 1.2 ± 0.6 | - |
| **CV2** | 0.8 ± 0.2 | 11.6 ± 3.3 |
| **CV3** | 0.5 ± 0.1 | 9.7 ± 1.0 |
| **CV4** | 0.4 ± 0.1 | 3.2 ± 1.6 |
| **CV5** | 0.5 ± 0.2 | 2.7 ± 0.8 |
| **CV6** | 0.3 ± 0.1 | 2.3 ± 0.4 |
| **CV7** | 0.2 ± 0.1 | 3.0 ± 0.9 |
| **CV8** | 0.5 ± 0.1 | 6.5 ± 0.7 |

**Supplementary Table S3.** Polymorphism sites in *PdoDAM1-6* genes among the three scaffolds.

|  |  | **scaffold1404-v.1.0** | | **scaffold1884-v.1.0** | | **scaffold31944-v.1.0** | |
| --- | --- | --- | --- | --- | --- | --- | --- |
| ***PdoDAM1*** | Size (bp) | 705 | | 705 | | 526 | |
|  | Polymorphisms | Position | Base | Position | Base | Position | Base |
|  | SNP | 213 | G | 213 | A | 34 | G |
|  | SNP | 582 | A | 582 | T | 403 | A |
| ***PdoDAM2*** | Size (bp) | 723 | | 717 | | 723 | |
|  | Polymorphisms | Position | Base/s | Position | Base/s | Position | Base/s |
|  | SNP | 10 | G | 10 | A | 10 | A |
|  | SNP | 222 | A | 222 | G | 222 | A |
|  | SNP | 237 | C | 237 | T | 237 | C |
|  | SNP | 246 | A | 246 | G | 246 | A |
|  | SNP | 402 | T | 402 | C | 402 | T |
|  | SNP | 439 | T | 439 | C | 439 | T |
|  | SNP | 470 | A | 470 | G | 470 | A |
|  | INDEL | 517-522 | ATGGTG | - | - | 517-522 | ATGGTG |
|  | SNP | 557 | C | 551 | T | 557 | C |
|  | SNP | 567 | A | 561 | G | 567 | A |
|  | SNP | 624 | C | 618 | A | 624 | C |
|  | SNP | 627 | A | 621 | A | 627 | G |
|  | SNP | 666 | C | 660 | A | 666 | C |
|  | SNP | 708 | A | 702 | G | 708 | A |
| ***PdoDAM3*** | Size (bp) | 708 | | 708 | | 708 | |
|  | Polymorphisms | Position | Base/s | Position | Base/s | Position | Base/s |
|  | SNP | 77 | G | 77 | C | 77 | C |
|  | SNP | 87 | G | 87 | C | 87 | C |
|  | SNP | 177 | T | 177 | C | 177 | C |
|  | SNP | 339 | A | 339 | G | 339 | A |
|  | SNP | 522 | C | 522 | C | 522 | G |
|  | SNP | 529 | C | 529 | C | 529 | A |
|  | SNP | 576 | C | 576 | C | 576 | T |
|  | SNP | 654 | C | 654 | T | 654 | T |
|  | SNP | 681 | A | 681 | T | 681 | T |
| ***PdoDAM4*** | Size (bp) | 693 / 678 * | | 693 / 678 * | | 693 / 678 * | |
|  | Polymorphisms | Position | Base/s | Position | Base/s | Position | Base/s |
|  | SNP | 24 | G | 24 | G | 24 | T |
|  | SNP | 93 | A | 93 | A | 93 | C |
|  | SNP | 95 | T | 95 | T | 95 | C |
|  | SNP | 179 | T | 179 | T | 179 | A |
|  | SNP | 192 | G | 192 | G | 192 | T |
|  | SNP | 211 | A | 211 | A | 211 | T |
|  | SNP | 354 | T | 354 | T | 354 | C |
|  | SNP | 355 | G | 355 | G | 355 | C |
|  | SNP | 356 | G | 356 | G | 356 | A |
|  | SNP | 358 | T | 358 | T | 358 | G |
|  | SNP | 359 | T | 359 | T | 359 | A |
|  | SNP | 393 | A | 393 | A | 393 | T |
|  | SNP | 509 | C | 509 | C | 509 | T |
|  | SNP | 519 | G | 519 | G | 519 | A |
|  | SNP | 535 | C | 535 | C | 535 | T |
|  | SNP | 537 | A | 537 | A | 537 | G |
|  | SNP | 665 | T | 665 | T | 665 | C |
| ***PdoDAM5*** | Size (bp) | 708 / 741 * | | 708 / 741 * | | 708 / 741 * | |
|  | Polymorphisms | Position | Base/s | Position | Base/s | Position | Base/s |
|  | SNP | 84 | C | 84 | C | 84 | T |
|  | SNP | 87 | A | 87 | A | 87 | C |
|  | SNP | 166 | C | 166 | C | 166 | G |
|  | SNP | 343 | C | 343 | C | 343 | A |
|  | SNP | 345 | T | 345 | T | 345 | A |
|  | SNP | 346 | C | 346 | C | 346 | G |
|  | SNP | 349 | A | 349 | A | 349 | G |
|  | SNP | 511 | G | 511 | G | 511 | C |
|  | SNP | 516 | G | 516 | G | 516 | A |
|  | SNP | 523 | A | 523 | A | 523 | G |
| ***PdoDAM6*** | Size (bp) | 717 | | 717 | | 717 | |
|  | Polymorphisms | Position | Base/s | Position | Base/s | Position | Base/s |
|  | SNP | 90 | T | 90 | T | 90 | G |

**Supplementary Table S4.** List of genes used in the phylogenetic analysis.

| **Species** | **Gene name** | **Accession code** | **Database** |
| --- | --- | --- | --- |
| *Actinidia deliciosa* | *AdeSVP1* | JF838212 | GeneBank |
|  | *AdeSVP2* | JF838213 | GeneBank |
|  | *AdeSVP3* | JF838214 | GeneBank |
|  | *AdeSVP4* | JF838215 | GeneBank |
| *Arabidopsis thaliana* | *AtAGL24* | AT4G24540 | TAIR |
|  | *AtSVP* | AT2G22450 | TAIR |
| *Euphorbia esula* | *EesDAM1* | ABY53594 | GeneBank |
|  | *EesDAM2* | ABY60423 | GeneBank |
| *Malus domestica* | *MdoDAM1* | MDP0000322567 | PlantTFDB |
|  | *MdoDAM2* | MDP0000259294 | PlantTFDB |
|  | *MdoDAM3* | MDP0000527190 | PlantTFDB |
|  | *MdoDAM4* | KT582789 | GeneBank |
|  | *MdoDAMb* | XP_028949089 | GeneBank |
| *Prunus armeniaca* | *ParDAM5* | QBY34764 | GeneBank |
|  | *ParDAM6* | QBY34765 | GeneBank |
| *Prunus mume* | *PmuDAM1* | BAK78921 | GeneBank |
|  | *PmuDAM2* | BAK78922 | GeneBank |
|  | *PmuDAM3* | BAK78923 | GeneBank |
|  | *PmuDAM4* | BAK78924 | GeneBank |
|  | *PmuDAM5* | BAK78920 | GeneBank |
|  | *PmuDAM6* | BAH22477 | GeneBank |
| *Prunus persica* | *PpeDAM1* | ABJ96361 | GeneBank |
|  | *PpeDAM2* | ABJ96363 | GeneBank |
|  | *PpeDAM3* | ABJ96364 | GeneBank |
|  | *PpeDAM4* | ABJ96358 | GeneBank |
|  | *PpeDAM5* | ABJ96359 | GeneBank |
|  | *PpdeDAM6* | ABJ96360 | GeneBank |
| *Pyrus pyrifolia* | *PpyDAM1* | BAI48074 | GeneBank |
|  | *PpyDAM2* | BAI48075 | GeneBank |
|  | *PpyDAM3* | BAM74166 | GeneBank |

**Supplementary Table S5.** Primers used in this study

|  | **Forward** | **Reverse** |
| --- | --- | --- |
| *PdoDAM1* | CCGTCTATTGAGTTGCAGCCA | GTAGCAGATTCAGATGACATGCCT |
| *PpeDAM1* | GGGGACGATGAAAATGACGAGGGAG | GTGGTGGAGGTAGCAGATTCAGAT |
| *PdoDAM2* and *PpeDAM2* | CAGTCAGCCAGCAGGAGAAGCAGCC | ACCAGCTCAGCTCCCTCAGT |
| *PdoDAM3* and *PpeDAM3* | ACCAGCTAAGGCAGACGATGA | GAGGGAGAGAGACTGAGAGCA |
| *PdoDAM4* | GATGAGTTGCTGAAGTTGGAACATCTG | CAGGTTACTTTCCCCAGGCCAC |
| *PdoDAM4.1* | ATGGCGATGTTAAGGGAGAGGAT | CGAACCGGTTAGCCCCAGTT |
| *PdoDAM4.2* | ATGGCGATGTTAAGGGAGAGGAT | CAAGAAAATTACGGACGCCCCA |
| *PpeDAM4* | TGTGGCACTTGAGAAAAAGGGA | CAGGTTACTTTCCCCAGGCCAC |
| *PdoDAM5.1* | GGACCTGCGCTTATGGATCA | TCCACTTCTTAACGCCCCAGTT |
| *PdoDAM5.2* | GGACCTGCGCTTATGGATCA | CCTCAACTGAAGCTTTCCCAGT |
| *PdoDAM5* and *PpeDAM5* | CCCCGAAACCCACGAACGAAGATG | CAGCACTGTTGCAGGTGGTG |
| *PdoDAM6* and *PpeDAM6* | TACTGGACCTGCGTTTGTGGAGCC | TGTTGCAGCTGGTGGAGGTGGCAATT |
| *TIP*-like | AGTTGTTGCTTGCTTGCTACTCAAG | GCACCAACAATCAAACCAATTGCGA |
| *SAP1*-like and *PpeSAP1* | ACACAGGCTTCCTCTACTCCATCTTT | GAACCCTCATTCCGAGACATTTATCAG |
| *S6PDH-*like and *PpeS6PDH* | CTACATGGCACGACATGGAAAAGAC | GGCGTAAGATAAGCAATCTCTGGTC |
| *RPG1*-like | TTCCAATCGGTGCCATATCTGGTAG | GAAGAGCCCCACGTTCATAAAACCA |
| *Peroxidase*-like | TCGCAATGGCCTGCTGTAACACAGA | CCGGTTTCAACTTCGGGTGGGGAT |
| AGL26-like | ACCACCTGAAGTCCTCCAAGATTG | GCTTCATACAAAGCAATGCCAACAC |
| actin-like | CTTCTTACTGAGGCACCCCTGAAT | AGCATAGAGGGAGAGAACTGCTTG |
| SAND-like | TCGTGGGTACCAGGAAAACGACAT | CCTGCTAGCTTGTGTTCATCTCCA |
